# Response of Plant Rhizosphere Microenvironment to Water Management in Soil- and Substrate-Based Controlled Environment Agriculture (CEA) Systems: A Review

**DOI:** 10.3389/fpls.2021.691651

**Published:** 2021-08-11

**Authors:** Bo Tan, Yihan Li, Tiegang Liu, Xiao Tan, Yuxin He, Xueji You, Kah Hon Leong, Chao Liu, Longguo Li

**Affiliations:** ^1^State Key Laboratory of Hydraulics and Mountain River Engineering, College of Water Resource and Hydropower, Sichuan University, Chengdu, China; ^2^Department of Hydraulic Engineering, College of Civil Engineering, Tongji University, Shanghai, China; ^3^Department of Civil, Architectural and Environmental Engineering, The University of Texas at Austin, Austin, TX, United States; ^4^Department of Environmental Engineering, Faculty of Engineering and Green Technology, Universiti Tunku Abdul Rahman, Kampar, Malaysia

**Keywords:** controlled environment agriculture (CEA), water management, rhizosphere microenvironment, microbe, root exudates

## Abstract

As natural agroecology deteriorates, controlled environment agriculture (CEA) systems become the backup support for coping with future resource consumption and potential food crises. Compared with natural agroecology, most of the environmental parameters of the CEA system rely on manual management. Such a system is dependent and fragile and prone to degradation, which includes harmful bacteria proliferation and productivity decline. Proper water management is significant for constructing a stabilized rhizosphere microenvironment. It has been proved that water is an efficient tool for changing the availability of nutrients, plant physiological processes, and microbial communities within. However, for CEA issues, relevant research is lacking at present. The article reviews the interactive mechanism between water management and rhizosphere microenvironments from the perspectives of physicochemical properties, physiological processes, and microbiology in CEA systems. We presented a synthesis of relevant research on water–root–microbes interplay, which aimed to provide detailed references to the conceptualization, research, diagnosis, and troubleshooting for CEA systems, and attempted to give suggestions for the construction of a high-tech artificial agricultural ecology.

## Introduction

The rapid expansion of modern cities has brought unprecedented challenges to sustainable development. It has caused urban complex diseases, such as environmental pollution and resource shortages (Despommier, 2011; Xie et al., 2017). While agroecology is suffering tremendous damage, requirements for the quality of agricultural products are getting higher, thus creating a contradictory situation within the current agroecological system (Orsini et al., 2013). In particular, with reference to European food safety policy, many countries and regions have stricter requirements for food safety and hygiene, labeling rules, regulations on plant health, control of pesticide residues, and food additives. Environmental issues bring people to contemplate the future of agriculture. As one of the solutions, high-tech agricultural technology is currently known as the manifestation of sustainable intensification (Pretty, 2018). It has taken the lead in large-scale development in developed regions, including the Americas and Europe. Through the construction of urban high-tech agricultural projects, one can solve the problems of climate change and urban food supply efficiently (Fatemeh et al., 2018; Fricano and Davis, 2019; Farhangi et al., 2020).

Controlled environment agriculture (CEA) is becoming a backup technology to cope with resource consumption and potential agricultural environmental deterioration in the future (Despommier, 2011). In CEA systems, the key parameters of production are artificially controlled. By controlling light, temperature, CO_2_, and humidity, indoor environments can become feasible for the growth of plants inside built-up spaces (Fatemeh et al., 2018; Farhangi et al., 2020). Various environmentally controlled structures can be classified as CEA systems, including soil-based CEA systems (e.g., high tunnels, greenhouses, growth chambers, and warehouse farming) and soilless cultivation (e.g., hydroponics, aeroponics) (Niu and Masabni, 2018).

Through long-term development and practices, CEA represents highly dependent modern agricultural technology. If a CEA system has more controllable environmental factors, correspondingly, it has a higher degree of closure and system integrity (Shi et al., 2009; Despommier, 2011; Hong et al., 2014; Wang et al., 2017; Fatemeh et al., 2018; Singh H. et al., 2020). The practice has shown that, under precise artificial regulation, water consumption can be reduced by 90%, and the yield can reach 20 times than that of incumbent agricultural production practices (Barbosa et al., 2015; Fatemeh et al., 2018). However, high-energy consumption, poor nutrient condition stability, the potential proliferation of pathogenic bacteria, and degradation of system productivity, etc., could be the mixture of uncontrollable factors that hampers CEA development (Hosseinzadeh et al., 2017; Niu and Masabni, 2018; Salazar-Moreno et al., 2020).

The popularization of CEA requires great effort, especially from developing countries where the substrate-based low-to-medium-cost CEA systems have not yet formed an industrial scale. The main technical difficulty lies in the scarcity of implementation standards for planting substrate management. The rhizosphere is the most valuable constituent of a CEA system (Zhang et al., 2019), and water is responsible for substrate decomposition, mass balance, and energy conversion. It is also vital for the microbial community (Singh et al., 2011; Arikan and Pirlak, 2016). At this stage, we propose that the first step is to establish an understanding of the nature of the rhizosphere microenvironment based on water management.

As growing cycles of replanting can be very short (e.g., less than 4 weeks for some leafy greens) in CEA systems, replant disease and negative legacy effects during certain planting generations can be significant due to nutrient consumption, rhizosphere bacterial community reshaping, and unfavorable rhizodeposition (Yuan et al., 2018; Sun et al., 2019; Yao et al., 2020). There were clear legacy effects from moisture regimes prior to planting on soil, specifically in terms of physicochemical properties, plant growth and nutrition, and the formation of microbial responsiveness (Cavagnaro, 2016). Hence, while CEA systems satisfy plant growth, proper management of substrate water still needs to improve by increasing the input of endogenous organic matter, reducing the demand for exogenous mineral nutrients, and enhancing beneficial biological activity (Jain et al., 2020). Water management is, therefore, a challenge with significant influence on the availability and sustainability of the planting substrate (Qin et al., 2019) that plays a vital role in both eliminating negative legacy effects and maintaining the long-term health of the rhizosphere for the whole system. Hence, water management is of great importance for successive planting generations (de Zeeuw et al., 2011; Poncet et al., 2015; Napawan and Townsend, 2016). The aim of water management in CEA in this review is to (1) regulate the availability of nutrients in the rhizosphere microenvironment, (2) regulate the physiological processes of plants, and (3) construct the microbial community structure for system benign output.

In this review, first, we summarize the advances and distribution of practical CEA systems worldwide, emphasize the characteristics of rhizosphere microenvironments and the role of water management in CEA systems, and analyze the influences on the physicochemical properties of the substrate, including aeration, solute dissolution, nutrient availability, transformation, and consumption. Next, we consider the effect of water content variation on the biochemical processes of the rhizosphere, address the interaction of root exudation and rhizosphere moisture stabilization, and discuss the rhizosphere water stress tolerance under water-limited conditions. Afterward, we analyze the influence of water content variation on microbial community structure and discuss the influences on microbial population, nutrient type, metabolism, and proliferation. Accordingly, we then summarize relative practice cases, showing that establishing a reasonable and stable rhizosphere microbial community structure is beneficial to the benign output of the CEA system. Then, we address the model characterization for microbial traits for microenvironment interaction and discuss the interplay of the abovementioned regulatory phenomena. Finally, we conclude by discussing the limitations and technical challenges of the current research on CEA systems, proposing two issues on the possibility and potential for future science and technology to improve water management of CEA, and offering suggestions about the construction of high-tech artificial agricultural ecologies for the future. The literature retrieval report related to this review was attached to Supplementary Material.

While we attempted to synthesize the available literature by summarizing results into practicable management methods, we acknowledge that there are many factors that may further affect the microenvironment that we were not able to introduce in detail, including root exudation patterns and responses in mixed communities, relationships between plant signal and microbial response, molecular mechanisms of host plants against pathogens, growth-promoting characteristics of endophytic bacteria and rhizobacteria, etc. (Ullah et al., 2019; de Vries et al., 2020; Williams and de Vries, 2020; Yadav et al., 2020).

Furthermore, our understanding of the response of rhizosphere microenvironments to artificial water management is hampered by the fact that there is only a very limited number of available studies on how water conditions in CEA relate to substrate physicochemical properties, root physiological processes, and rhizosphere microbiology; of those studies that do concern this topic, only a modest proportion focuses on controlled environments. In this review, we argue that an increased understanding of the complex feedback between water management and rhizosphere microenvironment evolution will pave the way for the conceptualization, construction, research, diagnosis, and troubleshooting of CEA systems.

## Concept and Characteristics of CEA Systems

### Understanding of the CEA System

Modern controlled environment agriculture has become an emerging form of land use in many developed regions (Sanye-Mengual et al., 2018), and the emergence is caused by the need to meet growing centralized demand for agricultural products and requirements for higher food security (Eigenbrod and Gruda, 2015). Advanced agriculture systems provide opportunities to improve food supply, the health of residents, the local economy, social integration, and environmental sustainability altogether (Orsini et al., 2013). An emerging CEA system has some notable characteristics: resource intensiveness, controllability, environmental fragility, high energy consumption, and high output.

Meanwhile, the CEA system usually has different manifestations. In vertical agriculture, plant growth substrates are strictly isolated and the system regulates nutrients to achieve clean, efficient, and high yield (Despommier, 2011; Fatemeh et al., 2018). In plastic greenhouse agriculture, the soil ecosystem is not completely isolated because the water, solutes, and microbes in the greenhouse soil still have interactions with the external environment, but the air composition is controlled, especially for some greenhouses with good airtightness (Shi et al., 2009; Hong et al., 2014; Wang et al., 2017; Singh H. et al., 2020). In closed hydroponic agriculture, plants are cultivated by using a mixture of nutrient salts and water instead of soil. The water is under treatment while circulation, therefore, the interaction between plant roots and the rhizosphere microenvironment is eliminated (Hosseinzadeh et al., 2017).

In a narrow sense, a CEA system is a set of agricultural planting facilities established in a specific enclosure. However, spatial isolation cannot accurately differentiate its intrinsic properties from traditional cultivation systems (Orsini et al., 2013). Therefore, a more specific definition could be, an industrialized agriculture system established in an independent space to maintain the continuous and stable regulation of plant growth factors through intensive management, thereby achieving optimal agricultural production and system sustainability (Eigenbrod and Gruda, 2015; Burchi et al., 2018).

### Advances and Distribution of Emerging CEA Practices Worldwide

**FIGURE 1:**
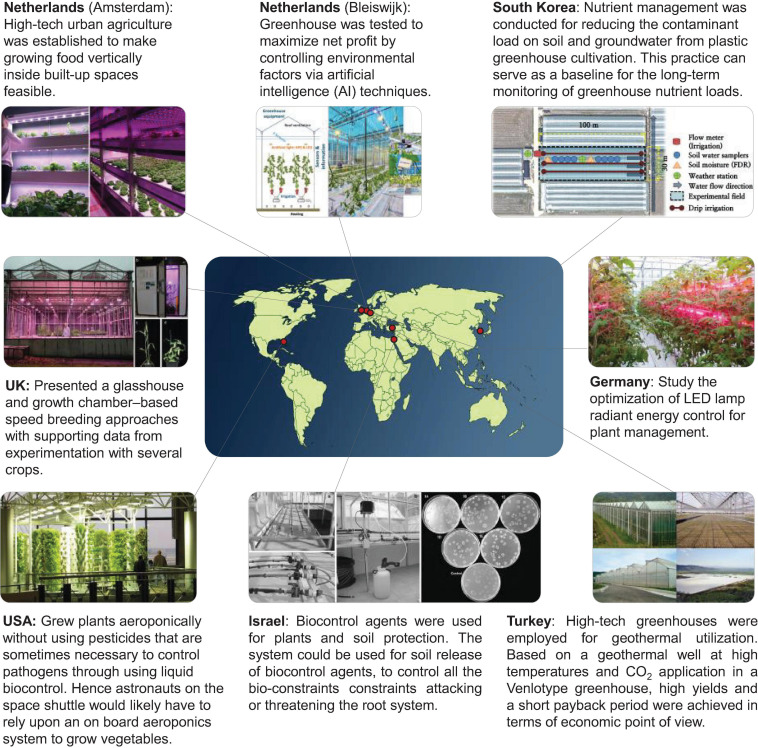
Exploration and application of controlled environment agriculture (CEA) systems worldwide. The figure compiled information based on the following references: Boari et al. (2008), Hong et al. (2014), Eigenbrod and Gruda (2015), Tuzel and Oztekin (2016), Ghosh et al. (2018), Lakhiar et al. (2018), Farhangi et al. (2020), Hemming et al. (2020).

In the context of the global agricultural revolution, the CEA system construction is meant to be combined with actual local conditions and social needs, and the functions of the CEA system are improving to serve the local food production (Napawan and Townsend, 2016; Clucas et al., 2018; Amato-Lourenco et al., 2020; Zulfiqar et al., 2020). Decades ago, in Israel, precision agriculture in greenhouses was employed for biological control (Boari et al., 2008). This approach allowed the irrigation system to be more compatible with the integrated treatment of biological control. In recent years, there has been a growing number of innovative treatments, using irrigation systems, such as the application of biosurfactants (Singh R. et al., 2020), generation of nanobubbles (Xiao et al., 2020), air injections (D’Alessio et al., 2020), and so on. In the Netherlands (Hemming et al., 2020), artificial intelligence (AI) algorithms and sensor data were used to determine climate set points and crop management strategies in greenhouse operations. Based on this technology, a greenhouse that could control ventilation, irrigation, heat, light, and CO_2_ was developed to maximize the net profit. As shown in Figure 1, at present, many representative emerging CEA systems in the world are mainly distributed in North America, Europe, the Middle East, etc., and their development orientation is toward integration into the urban context, aerospace engineering, and exploration into the integration of emerging AI and Internet of Things (IoT), and so on (Fatemeh et al., 2018; Lakhiar et al., 2018; Hemming et al., 2020; Halgamuge et al., 2021; Shuyu et al., 2021).

### Significance and Characteristics of the Soil- and Substrate-Based CEA Systems

Studies have shown that the development of CEA is driven by policy and economic factors (Hunold et al., 2016; Ghosh et al., 2018). On a global scale, however, the emerging CEA systems are currently incompatible with major food supplies due to high land prices and pollution in cities (Eigenbrod and Gruda, 2015). The soil- and substrate-based CEA systems, as a transitional form of traditional agriculture to agriculture industrialization, are integral parts of the agricultural supply chain in many parts of the world and are of central importance to research, technological improvement, and acceptance by the global agricultural economy. At the current stage of global CEA development, energy ratio and economic benefits are key factors (Farhangi et al., 2020; Hemming et al., 2020; Ntinas et al., 2020), while solid substrates have the advantages of low energy consumption, relatively high stability, and nutrient accumulation, which will have long-term existence in CEA development (Sanye-Mengual et al., 2018). Hence, compared with other forms of cultivation methods (the roots are in direct contact with the solution and air and do not adhere to solids), such as hydroponics and aeroponics, the use of solids as a growth substrate is still irreplaceable.

Considering the cost of the CEA system, managers always hope to improve its sustainability and expect to find more scientific approaches toward improving stability and resistance (Balafoutis et al., 2017; Al-Kodmany, 2018). A high-tunnel greenhouse is widely used in China (Shi et al., 2009). Research studies have shown that intensive production had a significant impact on soil and water quality. The rate and composition of fertilizers applied to vegetable plants were controlled for higher yield; meanwhile, it was equally important to protect the nutrient balance in rhizosphere soil and groundwater safety. In South Korea (Hong et al., 2014), the ecological safety of soil and groundwater was also closely considered during the implementation of plastic greenhouses. The substrate temperature is another factor that influences greenhouse cultivation. In Turkey, the CEA system (the main structure was a Venlo-type greenhouse, with 8 m width, 6 m gutter, and 7 m ridge height) took advantage of its geothermal resource by using heat exchanger-based heating systems on geothermal wells (Tuzel and Oztekin, 2016). Such heating systems enabled the CEA to obtain high yields and short payback periods in terms of an economic point of view for long-season production.

However, current pilots are quite scattered worldwide, and no universal technical specifications and standards have been formed, resulting in slow promotion and weak reproducibility (Shamshiri et al., 2018). To address these problems, soil- and substrate-based cultivation is emphasized in the following sections of this review. Since we discussed the relationship between water and crop plants, many crops are relatively tall and with large biomasses (such as maize); traditional hydroponics and aeroponics have not yet been applied to many food crops. On the other hand, since the water is treated in the recycling process, hydroponics and aeroponics are generally not involved in the concept of the rhizosphere microenvironment. Therefore, the CEA system discussed in this study refers specifically to soil- or substrate-based cultivation. With these considerations, the CEA mainly refers to soil- and substrate-based systems unless otherwise specified in this study.

### The Issue of Water Management for the Growth Media in CEA Systems

Comparing with the emerging CEA systems, the underlying logic for the establishment of soil- and substrate-based CEA is sustainability and low-resource consumption, which require inheriting the traits of rhizosphere microenvironments from natural soil-plant interaction. Water management is a basic yet efficient method for building a stable rhizosphere microenvironment (Gao et al., 2019). In terms of cultivation, the difficulty of CEA development lies in the management of growth substrates, nutrition, and irrigation (Orsini et al., 2013; Hong et al., 2014). Water content variation also affects physical and chemical properties and root exudation and then drives microbes to change their resource utilization strategies under different nutrient conditions (Preece and Penuelas, 2016). As a result of these changes in rhizosphere microenvironments, microbial populations and community structures can be determined.

The practice has proven that growth media substrates can significantly change the water status of their rhizospheres as compared to soil (Banitalebi et al., 2019; Videgain-Marco et al., 2020). These materials include a wide range of organic- and mineral-based substrates (mineral wool, peat, coconut fiber, lignite, straw, composted bark and wood fiber, perlite, vermiculite, sandy soil, clayey soil, etc.) (Gajc-Wolska et al., 2008; Słowińska-Jurkiewicz and Jaroszuk-Sierocińska, 2011). The physical properties of these materials are varied, and such discrepancies give a great scope for using multiple components for combination. Wider ranges of properties, such as volumetric density (30–1,400 kg⋅m^–3^), porosity (45–99% vol), water-holding capacity (-10 cm H_2_O, 15–85% vol), and air-holding capacity (–10-cm H_2_O, 20–80% vol), can also be obtained to adapt to a particular planting pattern (Lazny et al., 2021).

**FIGURE 2:**
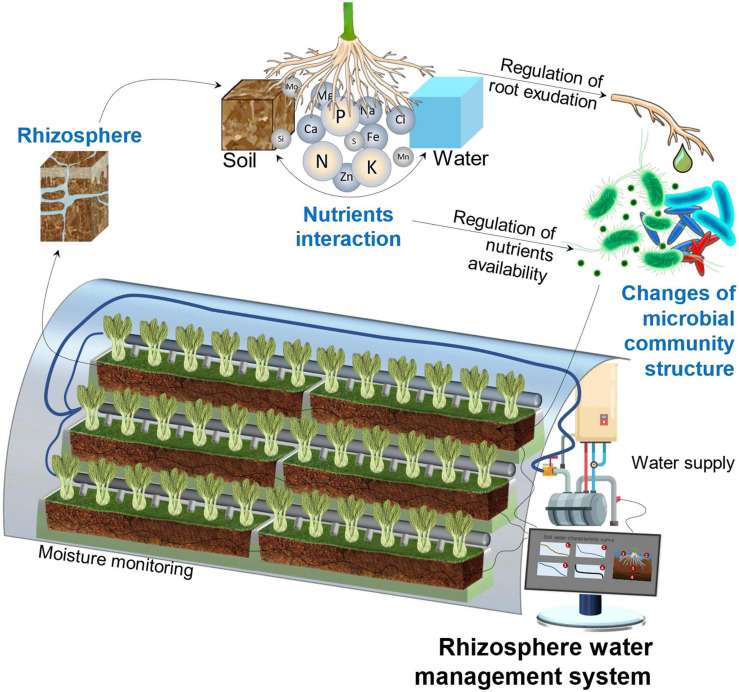
Important influential units of a CEA system under rhizosphere water management (drawn by the authors).

Compared with natural agroecology, fragility is the major challenge for the sustainability of substrate-based CEA, which means that the system is less resistant to adversity, pests, diseases, and pathogenic bacteria (Niu and Masabni, 2018) and, thus, has to rely on precise and intensive management to maintain stability. As shown in Figure 2, the rhizosphere is the most important area of a CEA system. It refers to the small volume of soil or substrate that is directly influenced by root exudations and associated microbes (Pii et al., 2015; Ahmadi et al., 2017). Different from the microbial abundance in the natural ecological environment, which is dominated by a diversity of local plant species and stable microbiomes (Qin et al., 2019), a CEA system depends on planned artificial regulation, such as substrate selection, water management, crop rotation, soil heritage, and inoculation of symbiotic bacteria. A well-managed rhizosphere has a higher microbial abundance and provides good nutrient accessibility with higher turnover rates (Herman et al., 2006; Landi et al., 2006; Holz et al., 2018a).

Water management for growth media, which is one of the most basic projects for agriculture, is particularly important for CEA systems. Based on supporting plant growth, balancing the input of endogenous organic matter and demand for exogenous mineral nutrients is the key to the optimization of system stability and low-resource consumption (Qin et al., 2019). It is crucial to reduce the maintenance cost brought about by substrate renewal on one hand, but, more importantly, to serve a long-term, sustained, and high-yield artificial agriculture system by constructing a specific rhizosphere microenvironment (Philippot et al., 2013; Qin et al., 2019). Furthermore, proper water management should focus on the availability of nutrients related to microbiology, as it is crucial to fostering the presence of beneficial microbes and reducing the level of pathogenic microbes, thus achieving system sustainability and benign output (Fierer, 2017; Degrune et al., 2019).

## Effect of Water Management on the Availability of the CEA Substrate *via* Affecting Its Physiochemical Properties

### Aeration and Solute Dissolution

In rhizosphere microenvironments, water content has a large influence on the physicochemical properties of the substrate. The overall resource utilization of the system is related to O_2_ concentration (Chen et al., 2019b; Li Y. et al., 2020), solute transport and diffusion (Nobel and Cui, 1992; Carminati et al., 2009; Ahmadi et al., 2017), and substrate decomposition of microbes (Tang et al., 2016; Zhang et al., 2020). A deeper understanding of its operating mechanism is the prerequisite for proper management.

In saturated substrates, pores are filled with water and the nutrients are sufficiently dissolved and supplied to plants for uptake. However, stagnant water brings about anaerobic conditions. At this stage, organic matter, rhizosphere exudates, and other substances are used for anaerobic decomposition (Tang et al., 2016; Zhang et al., 2020). A longer anaerobic period may cause major changes to the structure of the substrate microenvironment system because the anaerobic conditions are conducive to the growth of anaerobic bacteria. Anaerobic experiments have observed an increase in the abundance of methane-producing archaea and a significant increase in methane emissions (Miller et al., 2001; Bao et al., 2014). Therefore, an increase in water content is conducive to improving the activity of anaerobes and the utilization of substrates (Tang et al., 2016), but, from another perspective, it exacerbates the net loss of organic carbon, which needs to be evaluated by system managers.

When water content drops to field water-holding capacity (50–80% of the saturated water content), aerobic conditions are initially formed. In this case, large pores are filled with air, which is conducive to the diffusion of O_2_, and small pores are filled with water, which is conducive to the diffusion of soluble substrates. The soil or substrate emits a large amount of CO_2_ through heterotrophic respiration. At this time, aerobic metabolic activity reaches its maximum, while the CO_2_ flux is at the maximum (Zhou et al., 2014). In general, microbial activity at approximately moderate humidity (60% of water-filled pore space) is higher than activity at very wet or very dry conditions (Suseela et al., 2012).

As the substrate dries, the interconnection of pores promotes the formation of aerobic conditions. Meanwhile, roots shrink and partially detach from substrates, and air fills into the gaps between the roots and substrates. Consequently, the lower hydraulic conductivity induces the restriction of water and transport of nutrients to the roots and limits the activity of the rhizosphere microenvironment (Nobel and Cui, 1992; Carminati et al., 2009; Ahmadi et al., 2017). For substrates, solute transport and diffusion are reduced due to thinner water film and a more tortuous transfer path on a particle surface, thus limiting the rate of substrate diffusion to microbial cells (Stark and Firestone, 1995). Finally, concentrations of free ions in the residual solution increase, including calcium carbonate, sodium, potassium (K), phosphorus (P), and other redox-sensitive compounds (aluminum, iron, molybdenum, etc.) relevant to plants (Bouskill et al., 2016b).

Low water content causes a decrease in water potential in cells, thereby reducing hydration and activity in enzymes (Stark and Firestone, 1995), restricting the migration of enzymes for decomposers to decompose the substrate (Manzoni et al., 2012), thus inhibiting microbial activity. In general, a decrease in water content corresponds to a slowdown of biogeochemical processes in the rhizosphere microenvironment. Therefore, the rhizosphere microenvironment under water-saving measures undergoes resource redistribution, forcing microbes to change their way of resource utilization, such as carbon and nitrogen (N) utilization pathways (Schimel et al., 2007; Bachar et al., 2010).

### Nutrient Availability

Variation in water content is one of the greatest impacts on the rhizosphere microenvironment (Fierer, 2017); as a result, nutrient availability is determined. Considering the nutritional requirements of the CEA system, it is necessary for managers to focus on the nutrient content of the substrates. It is an economic and environmentally friendly approach to maintaining a sustainable nutritional supply through water management.

Compared with the natural agricultural environment, nutrient availability in the CEA system is more sensitive to water changes (Niu and Masabni, 2018; Shamshiri et al., 2018). In natural systems, water affects the dynamics of nutrient availability by altering the balance between the death and growth of organisms; thus, the overall balance can be relatively stable in the long term (Blazewicz et al., 2014). However, the CEA system is not an ecosystem in any case; it largely relies on artificial control. For optimal nutrient conversion to production, it is important to coordinate fertilization with water management, because nutrients must be in an available form before roots can absorb them (Holland et al., 2018). The dry substrate has difficulty in providing available nutrients because the substrate has great matric potential for nutrients, which makes it impossible to uptake by the roots (Somma et al., 1998; Vetterlein and Jahn, 2004; Jin et al., 2015). This part of the nutrients is the nutrient pool of the substrate and is retained by 0.1–1.5 MPa of matric potential (an approximate wilting point). When water content increases, the potential decreases; thus, nutrients can be released for roots to absorb, including NH_4_^+^, NO_3_^–^, H_2_PO_4_^2–^, HPO_4_^2–^, K^+^, Ca^2+^, Mg^2+^, SO_4_^2–^, BO_3_^3–^, Cl^–^, Cu^2+^, Fe^2+^, Fe^3+^, Mn^2+^, MoO_4_^2–^, Zn^2+^, etc. (Kim et al., 2009). In addition to ion availability, organic matter in CEA systems is a key nutrient factor in sustainable operation. Water content affects respiration by changing the O_2_ content, composition and activity of microbes, and utilization of substrates (Linn and Doran, 1984a; Williams, 2007; Zhou et al., 2014; Sierra et al., 2015), hence determining the decomposition of organic matter (Huang et al., 2016). The detailed transformation and consumption of macroelements, the complex interplay of microbial trophic type, substrate nutrition variation, and roots exudation in relation to water management are discussed in the following sections.

### Macroelements Transformation and Consumption

A sustainable CEA system must have “living” substrates instead of inert substrates like rock wool or perlite; thus, the CEA system has strict requirements for nutrient use efficiency, which is a water–fertilizer coupling problem (Wang et al., 2018; Rasool et al., 2020). Within the limited rhizosphere, the transformation and consumption of macroelements are very sensitive to water content variation (Liu et al., 2015; Koch et al., 2020). Water management is even more important than fertilizer management in certain water-deficient conditions (Epie and Maral, 2018). N, P, and K are the most important macroelements, and their occurrences and transformations exhibit different characteristics in water content dynamics with the participation of microbes (Dhaliwal et al., 2019).

#### Nitrogen

Nitrogen exists in soil or substrate systems in many forms and changes (transforms) very easily from one formation to another (Cameron et al., 2013). The main forms of N include organic N, NH_4_^+^-N, and NO_3_^–^-N. N is among the vital elements needed for plant growth. Since plants cannot use or take N directly from the air, uptake is through N forms that include ammonium and nitrate in substrates (Hachiya and Sakakibara, 2017). However, in the rhizosphere, their transformation process is related to nitrification and denitrification by microbes, while water is the key environmental factor to regulate this process and the transformation balance is closely related to rhizosphere water content variation (Chen et al., 2019b). The microbes involved are mainly ammonia-oxidizing archaea (AOA), ammonia-oxidizing bacteria (AOB), nitrite-oxidizing bacteria (NOB), etc. (Gleeson et al., 2010).

**FIGURE 3:**
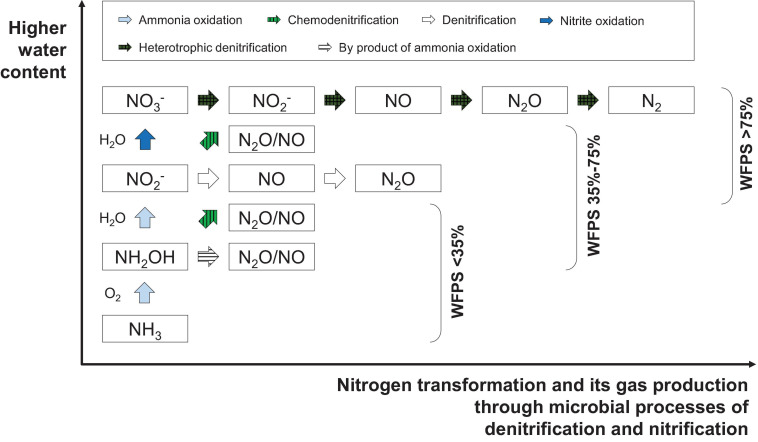
A schematic diagram of the main pathways of nitrogen (N) transformation and its gas production vs. varied water content in substrate. The figure is remade by compilation of the following research: Bateman and Baggs (2005), Gleeson et al. (2010), Kool et al. (2011), Zhu et al. (2013).

As for N transformation in the substrate, the importance of O_2_ as a controlling factor in regulating the magnitude and pathway of N has been recognized (Wrage et al., 2001); however, O_2_ concentrations are rarely measured in practice, and soil moisture content has generally been accepted as a measurable proxy for O_2_ availability (Zhu et al., 2013). Water-filled pore space (WFPS) is a widely used moisture indicator, as it provides integrated information about water content, total porosity, and O_2_ concentration of a soil or substrate system (Zhu et al., 2013; Qin et al., 2020). As shown in Figure 3, quick detection could be done by evaluating the N status based on WFPS and moisture content empirically or experimentally, because a gradual increase of the WFPS reflects the conversion of nitrification to denitrification. When the WFPS is 35–60%, O_2_ diffusion is favorable, the metabolic activity of aerobic microbes is at its most vigorous, and nitrification is dominant. Among them, net N_2_O emission is the lowest at approximately 40% WFPS (He et al., 2019), and more favorable conditions for nitrification is at 60% WFPS (Linn and Doran, 1984b; Skopp et al., 1990; Parton et al., 1996). When the WFPS is at 70–75%, O_2_ dissolution and diffusion rates decrease significantly, and it is impossible to provide O_2_ to aerobic microbes in time, which promotes denitrification and keeps the N_2_O emission rate at a high level (Orwin et al., 2010). Among them, 70–75% WFPS is a favorable condition for N_2_O emission. Denitrification consumes a large amount of NO_3_^–^ and allows N_2_O emission to reach its peak (Novosad and Kay, 2007; Qin et al., 2020). With increasing WFPS, O_2_ diffusion into the soil becomes restricted and the proportion of soil volume, which is anaerobic, increases. Due to the high mobility of NO_3_^–^, it may quickly diffuse into a substrate compartment with low O_2_ content, thereby providing substrate for biological denitrification. In addition, the massive production of NO_3_^–^ also promotes the volatilization of NH_3_. When WFPS is at 75–95%, the nitrification rate decreases significantly, and when WFPS is at approximately 80%, the denitrification effect could be at its utmost (Kool et al., 2011). When a substrate is nearly saturated (WFPS 90%), a large amount of NO_3_^–^ is lost and the production of N_2_O is mainly determined by NO_3_^–^ denitrification. When WFPS is at 100–125%, it becomes extremely anaerobic (Qin et al., 2020). Complete denitrification may occur, and NO_3_^–^ becomes the main substance for denitrification. As a result, N_2_O is completely converted to N_2_ under such anaerobic conditions (Zhu et al., 2013). This is because the anaerobic environment hinders the emission of N_2_O and promotes its further reduction to N_2_ (Qin et al., 2020).

#### Phosphorus

Phosphorus exists in various statuses and differs in its behavior and fate in soils or substrates (Hansen et al., 2004). Under water content variation, P has transformations among solution P (Sol-P), labile P (L-P), and non-labile P (NL-P). Sol-P is completely accessible for plants, but the bulk of P is virtually inaccessible, which can be described as NL-P. This fraction accounts for more than 90% of total P and is present as an insoluble and fixed form, including primary phosphate minerals, humus P, insoluble phosphate of calcium, iron and aluminum, and P fixed by hydrous oxides and silicate minerals (DeLonge et al., 2013). L-P is presented in phosphate precipitations and is held on substrate surfaces. It is also in rapid equilibrium with Sol-P. Consumption of Sol-P disturbs the equilibrium between Sol-P concentration and the L-P pool at a solid phase, which leads to supplementation for Sol-P (Bünemann, 2015).

Phosphorus in soil or substrate is mostly immobile and unavailable to plants and is further restricted when water availability is limited (Somaweera et al., 2017). Thus, the water content can be important to determining the P bioavailability and net primary productivity in planting systems (DeLonge et al., 2013). In general, higher water content is beneficial for the release of L-P and the improvement of P bioavailability, while, in saturation, reduction *via* anaerobic conditions may contribute to Sol-P release and result in the highest Sol-P concentration at wet extremes (Cournane et al., 2010).

The change of L-P content also follows a similar rule as Sol-P. Takahashi et al. (2016) found that L-P increased steadily when the water content was higher than the standard level (1 kg/kg) in incubated growth substrate. Epie and Maral (2018) suggested that L-P is best for root development, tillering, and growth when the water content is more than 75% field capacity; however, P availability is greatly reduced when the water content is less than 30% of field capacity. At this point, the effect of fertilization is limited by water stress. Yang et al. (2009) indicated that an ideal L-P content was obtained at 50% of field capacity, as it was suitable for substrate phosphatase to enhance activity, and thus promoted the release of L-P from NL-P.

As irrigation after extreme drying, L-P content increased significantly; this phenomenon was found in Styles and Coxon (2006); DeLonge et al. (2013), and Sun et al. (2018). But it has to be noted that water content is positively related to L-P dissolution; meanwhile, it was regulated by microbial activities. Vandecar et al. (2011) found a delayed response of L-P release under high-water content (50–66%), while DeLonge et al. (2013) indicated that large pulses of water input may facilitate L-P release, but the process has an 8 days delay. Microbial activities play an important role in P mineralization and immobilization and consequently affect L-P supplementation and depletion. It is clear that wet-dry circulation affects L-P content *via* the alternation between community composition and acceleration of organic matter decomposition, thus enhancing P mineralization (Sun et al., 2018). Similar results demonstrated that microbial immobilization of P was stimulated initially; however, a time lag of up to 10 days was found due to subsequent mineralization (Campo et al., 1998).

Altogether, how does L-P release largely depend on antecedent and current substrate moisture conditions (Howard and Howard, 1993; Yuste et al., 2007). L-P can be increased in the short term by drying and rewetting, with its effect dependent on both the size and timing of water management (DeLonge et al., 2013).

#### Potassium

Potassium is another primary nutrient required by plants. K is found within plant cell solutions and is used for maintaining the turgor pressure of the cell (meaning it keeps the plant from wilting) (Abd El-Mageed et al., 2017; Abd El-Gayed and Bashandy, 2018). In addition, K plays a role in the proper functioning of stomata (cells located at the bottom of leaves that open and close to allow water vapor and waste gases to escape) and acts as an enzyme activator (Xu et al., 2020). In a given substrate, total K is almost certain because it depends on the presence of K, which bears primary and secondary minerals, namely, fixed or mineral K (Ghiri and Abtahi, 2011; Škarpa and Hlušek, 2012). Hence, managers are more concerned about how water content variation affects the transformation between soluble K (Sol-K) and exchangeable K (Ex-K).

Exchangeable-K is the major bioavailable form of K in substrates. There is rapid equilibrium between Sol-K and Ex-K, which can be described by the Gapon equation (Beckett and Nafady, 1967). The consumption of Sol-K at a root-solid matrix interface causes a readjustment of Ex-K to satisfy the equilibrium equation as mentioned above, releasing more Ex-K into solution, thereby buffering Sol-K against consumption.

With regard to Sol-K, higher water content is conducive to K dissolution while also contributing to K dilution, depending on the substrate components that adsorb K. Research from Abd-Elrahman and Taha (2018) showed that humates and sulfates have the strongest ability to hold Sol-K because they prevent K^+^ ions from leaching, owing to the influence of multiple functional groups including carboxyl, phenol, and hydroxyl that, in turn, contribute to K^+^ binding (Wang and Huang, 2001). Marchuk and Marchuk (2018) demonstrated that a high level of Sol-K has deleterious effects on the structural stability of a growth matrix, while the fraction of clay minerals could decrease cation exchange capacity and increase a mineral fraction of K, resulting in K fixation as a non-exchangeable form. As Ex-K diffusion and Sol-K are consumed, an ever-widening zone of K consumption spreads out from the root surface, leading to the development of a rhizosphere several millimeters in radius (Kuchenbuch et al., 1986; Hamoud et al., 2019). Therefore, the amount of K is closely related to the cation exchange capacity of the substrate.

In practice, Abd-Elrahman and Taha (2018) showed that soils amended with either humate or sulfate under 50% of the irrigation requirement recorded the highest increases in the fraction of Ex-K; however, increasing the irrigation water level from 75 to 100% led to a significant reduction in the Ex-K fraction, suggesting that the increasing level of water irrigation seemed to be of no further significant effect on the Ex-K content. Higher water content does not increase Ex-K content, and this result also applies to longer-term effects, as consistent results were found in the study by Škarpa and Hlušek (2012). As with the importance of Sol-K and Ex-K, fixed or mineral K is the K source for sustained supply. Ghiri and Abtahi (2011) suggested K-bearing minerals could be considered as the K pool; meanwhile, intentionally, K fixation by wetting and drying treatment could also be a practical method for conservative planting.

## Effect of Water Management on Root Physiological Processes in CEA

### Root Growth and Exudation

Root exudates are fluids emitted through roots. These substances influence the rhizosphere around roots to inhibit harmful microbes and promote plant growth (Williams and de Vries, 2020). Root exudates contain a wide variety of molecules that are released into soil (Bobille et al., 2019). They act as signaling messengers that allow for communication between microbes and roots (Calvo et al., 2017). In CEA systems, rhizosphere exudation has more significance because the rhizosphere is bounded; hence, roots, microbes, exudates, and all sensitive substances are squeezed into the limited volume of a substrate cube. Thus, the impact of water changes is higher than in the natural environment (de Vries et al., 2020).

Plants release a large part of their photosynthetic products into soil or substrate through rhizodeposition, including low-molecular-weight compounds such as polysaccharides, amino acids, and organic acids (Fischer et al., 2010), and high-molecular-weight compounds such as mucoid biopolymers (Ahmadi et al., 2017). Most of the low-molecular-weight rhizosphere exudates are released from the growing tips of roots (Jones et al., 2009; Pausch and Kuzyakov, 2011). Root elongation is sensitive to water content variation and has an important influence on rhizodeposition due to major modification of the length and velocity of the exuding root zone (Sharp et al., 2004). An increase in rhizosphere water content enhances diffusion of exudate and increases its microbial decomposition (Holz et al., 2018a), while the diffusion has a strong influence on exudate distribution and the root exudation rate (Jones et al., 2004). The release and the diffusion capacity of exudates directly affect carbon distribution in the rhizosphere. Meanwhile, decomposition of rhizosphere exudates, root hair biomass, and adsorption capacity of microbes also affect the rhizosphere carbon content (Kuzyakov et al., 2003; Jones et al., 2009; Holz et al., 2018b).

However, limited irrigation quantities, a common water management measure in CEA systems, may cause water stress and initially promote redistribution of recently assimilated carbon, transfer it to roots, and synthesize rhizosphere exudates, whereas they may lead to a decrease in exudation intensity and ultimately weaken rhizodeposition (Sharp et al., 2004). They may affect the rhizodeposition process and change solute composition in the growth substrate in the long term.

### Rhizosphere Allelopathy

Rhizosphere exudates are also known as allelochemicals and can have beneficial (positive allelopathy) or detrimental (negative allelopathy) effects on rhizosphere microenvironments (Scavo et al., 2019). Maintaining the beneficial rhizosphere allelopathy and reducing the allelochemicals phytotoxicity is of central importance. The exudates of allelochemicals are responsible for the recruitment of beneficial microbes through the alteration of the rhizosphere microenvironment, thus mitigating unfavorable conditions (Li et al., 2014; Holz et al., 2018a; Naylor and Coleman-Derr, 2018).

Rhizosphere microbes are inseparable from plant rhizosphere allelopathy because the secreted allelochemicals are accepted as the energy source for microbes (Holz et al., 2018a); meanwhile, the allelochemicals play a role in communicating with rhizosphere microbes as signal compounds (Shah and Smith, 2020). Rhizosphere microbes give different kinds of feedback to plants under various nutritional conditions, while water is the key to regulating the process. Generally, the rhizosphere microenvironment tends to recruit microbes that can produce plant hormones when the substrate is enriched with minerals and nutrients under water sufficiency, which is known as the eutrophication state (Pascault et al., 2013; Hartmann et al., 2017). On the contrary, insufficient rhizosphere water leads to the formation of oligotrophic conditions. Taking rhizosphere allelopathy into account, one could ascertain that the significance of water management is the opportunity to take advantage of the recruitment effect and achieve proper mineralization, nutrient dissolution, and plant absorption (He and Dijkstra, 2014).

To improve water use efficiency in CEA systems, deficit irrigation is commonly employed. Declining water content causes changes to plant physiology and biochemistry. Significant impact lies in the change of substrate pH, root morphology, the total amount of carbon input, and the rhizosphere exudates (including soluble sugar, organic acid, mucilage, enzymes, and exfoliated cells, etc.) (Grierson and Adams, 2000; Marschner et al., 2005).

As with the changes in solution concentration, water deficiency is, essentially, a kind of osmotic stress on plant physiology. On the other hand, the bulk of situations of water deficiency increases enzyme activity during plant growth periods; meanwhile, it increases the concentration of organic acids in root exudates, thereby contributing to drought tolerance. Under water stress, the rhizosphere microenvironment of corn (*Zea mays* L.) has increased protease, catalase, alkaline phosphatase, and invertase activities. Osmotic stress increases the concentration of malic acid, lactic acid, acetic acid, succinic acid, citric acid, and maleic acid in root exudates (Song et al., 2012). Water deficiency enriches the root exudates of barley (*Hordeum vulgare* L.) with more proline, K, and phytohormone, which play important roles in promoting root growth osmotic protection and stress signal transduction (Calvo et al., 2017). Studies also showed the dependence of the microbial communities on activities of protease, urease, and phosphatase; these changes in substances are results of the rhizosphere allelopathy regulated by water (Marschner et al., 2005).

Water-stressed rhizosphere allelopathy could be an opportunity for one to make good use of it. Rhizosphere N-fixing bacteria have a higher N-dissolving ability under water shortage conditions, which is an approach to enhance plant growth (Knoth et al., 2014). Meanwhile, one can change the process of plant carbon assimilation, distribution, and deposition in the rhizosphere (Holz et al., 2018a), as well as the regulation of N mineralization (Akter et al., 2018). Detailed practical cases of water management with regard to the microbiome and production are discussed in Section “Microbial *C*ommunity.”

### Rhizosphere Physiological Adaptation

Plants can adapt to varied rhizosphere hydro-environments by nature (Ahmed et al., 2018) because the rhizosphere exudation is responsible for the adjustment of plants to substrate moisture, especially when the water content is undergoing wet-dry circulation (Carminati et al., 2010; Ahmed et al., 2016). The exudate, commonly found to be mucilage, plays an important role in substrate moisture regulation. The exudation intensity is largely affected by water content, while, in turn, exudates affect soil or substrate hydrophilicity, thereby changing the moisture status, which is a unique process in rhizosphere microenvironments (Palta and Gregory, 1997; Sanaullah et al., 2012). In CEA systems, mucilage is easily spread throughout the planting substrates because the substrate cubes are generally kept to a minimum size. Hence, the mucilage is more significant for wettability regulation and water retention in the limited volume of a substrate cube.

**FIGURE 4:**
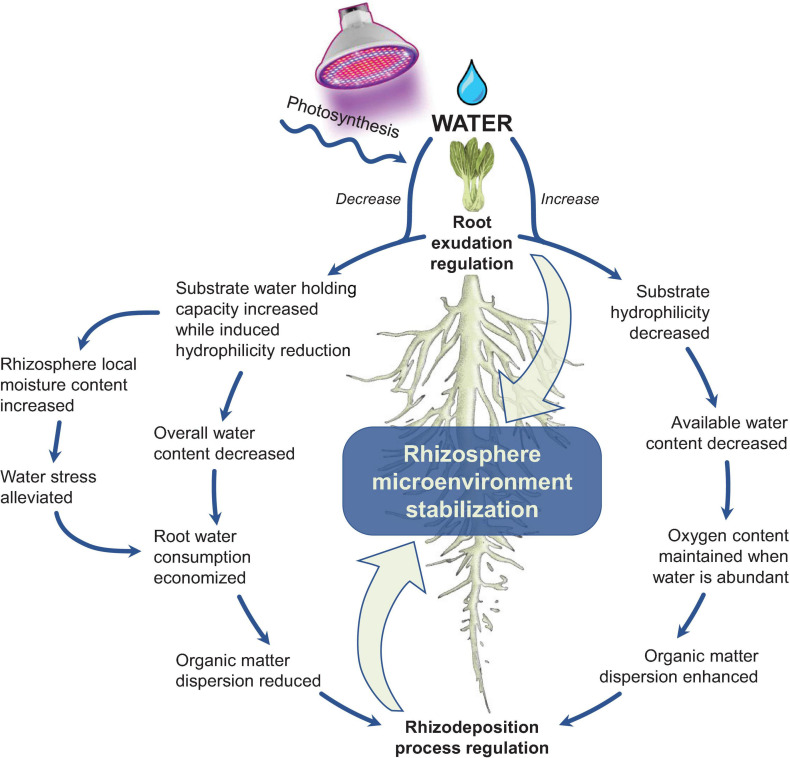
A mechanism of rhizosphere microenvironment stabilization under water content variation (drawn by the authors).

As shown in Figure 4, continuous mucilage exudation is a kind of self-compensation under water stress. The mucilage increases the local moisture content in the root direction and ultimately compensates for the negative impact of water deficiency. It is a strategy to maintain rapid diffusion of exudates and high microbial activity (Holz et al., 2018a). The influence of rhizosphere exudates on the rhizosphere microenvironment starts from the contact part between root sheath and substrate. The root sheath has several important functions for water and nutrient absorption, especially under water stress (Hsiao and Xu, 2000). This is because the root sheath keeps the root system in contact with the substrate during the drying process, thereby enhancing the hydraulic connection between the root system and the substrate and creating a rhizosphere microenvironment that is compatible with water (Drenovsky et al., 2004; Kuzyakov and Blagodatskaya, 2015). Under water-deficient conditions, roots secrete polysaccharide mucilage to preserve relatively more water, but the mucilage reduces the substrate hydrophilicity. This, in turn, reduces water flux in the rhizosphere, and thereby reduces water consumption by roots (Zarebanadkouki and Carminati, 2014). When the rhizosphere is subsequently wetted again, the water content will be lower than that of the blank substrate because of the previously reduced hydrophilicity. This mechanism ensures the relative stability of the rhizosphere microenvironment during alternation of wetting-drying (Read et al., 1999), which has also been proved experimentally.

The mucilage secretion strongly affects the biophysical properties of the rhizosphere, which determines the ability of roots to extract water and nutrients from its growing substrate. The rhizoligand is a mucilage analog (such as the commercial surfactant of AC 1820 acrylate copolymer) that is defined as an addictive substance that increases the wettability of the rhizosphere and links the mucilage network to main intimate contact with the root surface. Ahmadi et al. (2017) used the exogenous rhizoligand to demonstrate the influence of rhizosphere exudates on substrate hydrophilicity during the wetting-drying cycle. It was found that the rhizoligand improved hydrophilicity and enhanced the communication between the rhizosphere microenvironment and plants, thereby making the root sheath more developed. Meanwhile, the activities of chitinase, sulfatase, and β-glucosidase were 4, 7.9, and 1.5 times greater, respectively, and biomass was 1.2-fold that of water-irrigated plants. By adjusting hydrophilicity, this approach harnesses water availability without using conventional irrigation methods (Ahmed et al., 2016; Ahmadi et al., 2018).

In controlled environment agriculture systems, water deficiency or moderate drought may be an approach to enhance the mass and energy utilization efficiency of the system (Sposito, 2013; Ahmed et al., 2018). Owing to the small rhizosphere space and concentrated allelochemical substances as described above, self-stabilization of moisture in the microenvironment can be fully utilized to achieve better results than traditional irrigation. One can realize optimized economical resource input by fully exploiting the potential instead of simply satisfying the greatest physiological needs of plants (Qin et al., 2019), which could be an innovative concept for CEA management.

## Effect of Water Management on Rhizosphere Microbiology in CEA

### Microbial Physiology

System designers of controlled environment agriculture need to guide the rhizosphere microbiology to a beneficial and sustainable status (Colla et al., 2017). In terms of water management that acts upon rhizosphere microbial physiology, the main impact, in effect, is on carbon catabolism and cellular osmosis regulation (Vurukonda et al., 2016; Rajkumar et al., 2017). The regulatory significance lies in the regulation of hydrolytic enzymes and osmolytes production, which, in turn, influences the overall pattern of resource utilization (Blazewicz et al., 2014; Naseem et al., 2018; Sammauria et al., 2020).

Water saturation is favorable for facultative anaerobe to enhance substrate respiration and enzymatic degradation, which further increases the labile carbon fraction *via* carbon speciation. For instance, carbohydrates are particularly important in the carbon catabolism of a microbial community (Tate, 1979; Wilson et al., 2011; Fierer, 2017). It is clear that water saturation creates a nutrient-rich microenvironment and brings microbial proliferation; however, water deficiency can still stimulate the proliferation of oligotrophic bacteria in the dry rhizosphere (Naylor and Coleman-Derr, 2018); meanwhile, water use efficiency can be improved under relatively nutrient-poor conditions (Enebe and Babalola, 2018), despite the resource limitation for most microbes. On the other hand, as for the eutrophic microbes, water deficiency reduces microbial activity through dehydration and substrate limitation, and reduces the microbial metabolic process (Stark and Firestone, 1995); consequently, the formation of rhizosphere nutrients is determined.

**FIGURE 5:**
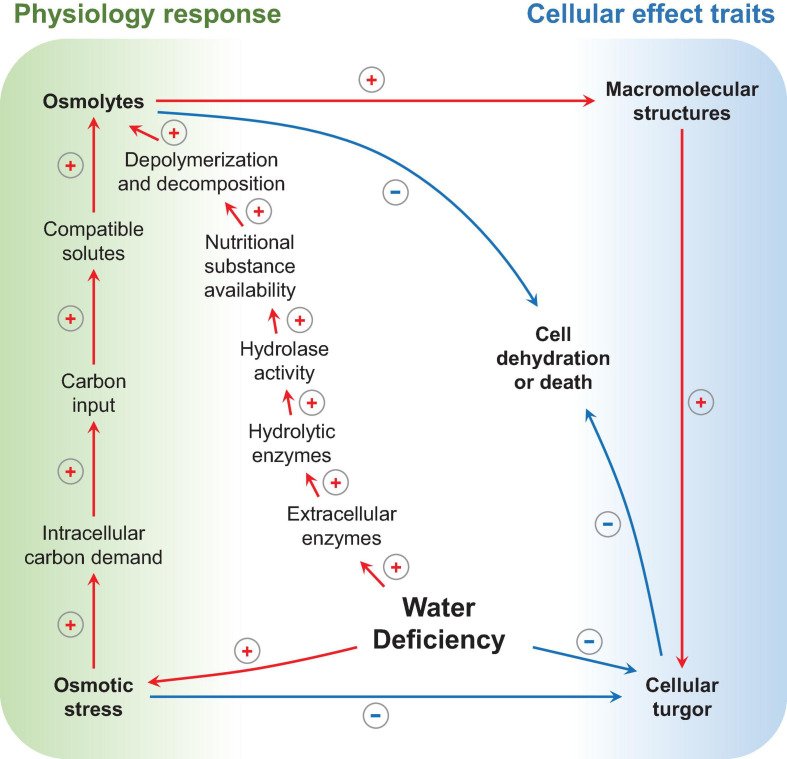
Microbial physiology response and cellular effect traits under water deficiency. Arrows indicate influence processes, positive red signs represent increase or enhancement, and negative blue signs represent reduction (drawn by the authors).

As shown in Figure 5, microbes under varied nutritional conditions release extracellular enzymes that can regulate depolymerization and decomposition (Bouskill et al., 2016b; Igalavithana et al., 2017), thereby mediating the overall circulation rate of a nutritional substance (mainly carbon and N) in the rhizosphere (Frossard et al., 2000; Schimel and Bennett, 2004). For example, when the rhizosphere undergoes water deficiency, changes occur in the functional potential of microbial communities that are concomitant with an increase in hydrolase activity (Alster et al., 2013). As is exhibited from the data on functional gene regulation, the genes-encoding extracellular enzymes that degrade chitin, cellulose, lignin, pectin, and enzymes involved in hemicellulose (xylose) catabolism were of higher relative abundance in water deficiency; meanwhile, the specific activities of the corresponding classes of enzymes were also higher (Bouskill et al., 2016b).

Osmotic stress is the driving factor in the physiological processes depicted above. Specifically, as substrate dries and water potential drops, cells must accumulate solutes to reduce their internal water potential to avoid dehydration or death (Schimel et al., 2007). Hence, the synthesis of osmolytes is necessary, and a large amount of carbon input is required. Under this circumstance, it was found that, as a response to osmotic stress, the intracellular carbon demand and production of compatible solutes increases (Bouskill et al., 2016b). For bacteria, amino compounds are typically used as osmolytes, such as proline, glutamine, and betaine (Csonka, 1989); while, for fungi, polyols, such as glycerol, erythritol, and mannitol, are used (Witteveen and Visser, 1995).

These physiological performances and adaptations are manifested in a dynamic process; microbes maintain cellular turgor and protect macromolecular structures by using osmolytes; meanwhile, these osmolytes regulate hydrolytic enzymes activity to acquire carbon for osmolytes synthesis and, ultimately, achieve the balance of carbon consumption and rhizodeposition (Welsh, 2000; Bouskill et al., 2016a; Vurukonda et al., 2016; Rajkumar et al., 2017; Naseem et al., 2018; Hartman and Tringe, 2019; Teijeiro et al., 2020).

### Microbial Community

Different substrate configurations affect microbial traits, and a “living” substrate could retain stable and beneficial microbial communities. Fresh and easily degradable organic matter in the substrate stimulates microbial growth and serves as an energy source for microbes to synthesize extracellular enzymes that are capable of degrading recalcitrant organic matter, thus facilitating mineralization. This is based on the way microorganisms live in the substrate that was explained by the “co-metabolism” theory (Kuzyakov et al., 2000; Wang et al., 2015). Although we did not find a specific case study on a microbial community that was affected by varied substrates, there is emerging consensus on which variables are most likely to have marked effects on the microbial community.

An optimized ratio of substrate carbon/N for microbial mineralization is believed to be around 20, which is calculated by dividing the microbial carbon/N ratio (10) by the carbon assimilation yield of microbial biomass (0.5) (Recous et al., 1995; Manzoni et al., 2010). However, with the consumption of nutrients, the microbial community develops in different directions; not surprisingly, it generally does not lead to desired yield and sustainability if the dynamic of the microbial communities is underestimated. Fei et al. (2008) showed a notable increase in bacteria and actinomycetes and a significant decrease of fungi in surface soil-based greenhouses; that is, the ratio of bacteria to fungi increased. As the substrate was used for a long time, the microbial biomass showed a downward trend. In agronomic practices by Bonanomi et al. (2017), soil with disinfestation treatments was used as the planting substrate for plastic tunnel farming systems. Mulching films were employed as a combination with microbial consortia, containing beneficial microbes (i.e., antagonistic fungi of the genus *Trichoderma*, mycorrhizal fungi of the genus *Glomus*, and the plant growth-promoting bacterium *Bacillus subtilis*). The application of beneficial microbes can indirectly increase water use efficiency by controlling soilborne pathogens and significantly increase root mycorrhizal colonization compared with untreated controls in all cropping cycles.

**FIGURE 6:**
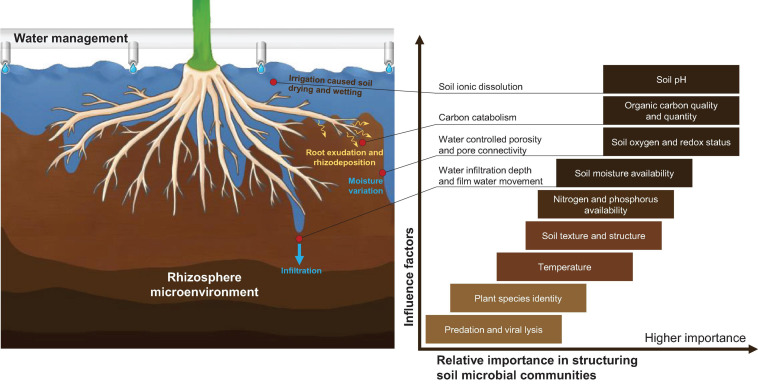
The importance of water management in rhizosphere microenvironments and the factors that structure microbial communities. The box color qualitatively indicates the current understanding of the specific effects of certain factors on microbial communities; darker shades highlight reasonably well-understood factors. The figure is remade using the compilation of the following research studies: Schimel et al. (2007), Lauber et al. (2009), Kuramae et al. (2011, 2012), Okegbe et al. (2014), Maestre et al. (2015), Prober et al. (2015), Fierer (2017).

We hoped to understand the link between the microbial community and the substrate parameters. As shown in Figure 6, among multiple factors, in addition to pH, water content, quality and quantity of organic carbon, and the redox state are the most significant factors that have notable influences on the structure of microbial communities (Schimel et al., 2007; Lauber et al., 2009; Kuramae et al., 2011, 2012; Okegbe et al., 2014; Maestre et al., 2015; Prober et al., 2015; Fierer, 2017).

The driving factors that lead to the difference between CEA planting substrate and field soil could be varied. Due to the complexity of the natural soil system, measurements of bulk soil (ectorhizosphere) properties do not necessarily capture the microscale variations in soil properties that may drive spatial variation in soil microbial community composition (Fierer, 2017). A broad range of different microbial habitats coexists in field conditions; meanwhile, microbial communities and microbial taxa are preferentially associated with different surface vegetation. This is true for many mycorrhizal fungi, fungal plant pathogens, and some N-fixing bacteria (for example, *Rhizobium* spp.), which, typically, only associate with specific plant species. However, in CEA systems, the microbial community can be significantly regulated, which provides a basis for the application of growth-promoting bacteria (PGPB). A cluster of bacteria that colonize the root of the plant rhizosphere is termed the “PGPB” (Dhayalan and Sudalaimuthu, 2021). The role of PGPB in plant growth is of importance to water regulation because the PGPB could induce a plant to tolerate water deficit conditions *via* colonies in rhizospheres and endorhizospheres, and it could provide a wider range for water regulation in the system. Sandhya et al. (2010) proved that the plant biomass was enhanced through the inoculation of *Pseudomonas putida* under drought conditions. Armada et al. (2014) found that the concentration of proline in the *Lavandula* shoot was increased by inoculation of *Bacillus thuringiensis*, thereby promoting plant growth.

In controlled environment agriculture–planting substrates, moisture and aeration could be the most significant contributors to determining microbial composition. Water condition decides the O_2_ content and nutrient availability (Drenovsky et al., 2004) and reshapes the community structure of eutrophicor oligotrophic microbes; as a result, the nutritional type of the microbes is determined (Hedenec et al., 2018). When water deficiency occurs, it is nutrient-poor but oxygen-rich in the substrate; thus, microbes die or enter dormancy and the overall activity tends to decrease. However, it sets up a stage for aerobic bacteria and/or oligotrophic bacteria (Blazewicz et al., 2014; Armstrong et al., 2016).

The different behaviors of Gram-positive bacteria and Gram-negative bacteria are a good example showing the community structural changes under water deficiency. Gram-positive bacteria are metabolically more “tenacious,” as they can use recalcitrant compounds to produce extracellular enzymes (Treseder et al., 2011; Naylor and Coleman-Derr, 2018). *Actinomycetes*, the oligotrophic bacteria under the phylum Gram-positive bacteria, are metabolically versatile. They can degrade complex organic compounds, maintain high-carbon utilization efficiency, and form spores and filaments through cellular modification (osmotic protectants, dormancy) (Hartmann et al., 2017). These abilities ensure their survival and even dominance in the substrate with low-hydraulic connectivity and nutrient limitation (Wolf et al., 2013). Cell walls may render microbes more resistant to water potential decrease; for example, Gram-positive bacteria can survive in stress by producing strong, thick, and interlinked cell walls of peptidoglycan (Schimel et al., 2007). Another strategy for microbes to withstand stress is sporulation, which is considered a potential factor in observing the trend of abundance. Many genera in Gram-positive phyla are known as sporophytes, while the Gram-negative phyla have mostly lost the ability to sporulate during evolution (Tocheva et al., 2016).

Water content variation reshapes the microbial community structure significantly, while it has a minor effect on diversity (Bachar et al., 2010; Blazewicz et al., 2014). On the other hand, the different behaviors of bacteria and fungi under water deficiency merit attention, especially under long-term water stress. Bachar et al. (2010) showed that bacteria abundance had a decreasing trend with the degree of water deficiency; however, bacteria diversity had less relevance to water content. Acosta-Martinez et al. (2014) showed that 10 months of severe drought caused the fungal diversity index and OTUs to increase more than bacteria, and found that *Proteobacteria*, *Actinobacteria*, *Chloroflexi*, and *Nitrospirae* have higher abundance. While significant progress has been made in exploring the relationship between how water and nutrition shape microbial communities, the extent to which water affects rhizosphere plant-microbe interactions is still elusive. It remains to be seen which of the detected correlations will prove to be significant for microbial diversity and structural composition, and which will prove redundant (Aung et al., 2018; Qin et al., 2019; Jain et al., 2020).

As shown in Table 1, for the benign output of the system, there have been many attempts for different crops. To be sure, proper water management is conducive to the benign output of an agricultural system. There are constantly increasing practical experiences on this issue. The method of negative pressure irrigation is a water supply technology for water-saving and fertilizer utilization efficiency improvement, which emits water through a porous ceramic tube embedded in the rhizosphere. This method can consistently supply water, depending on the water consumption of the plants and soil tension (Long et al., 2018). Zhao et al. (2019) employed negative pressure irrigation to supply water in relation to soil matrix tension and water consumption during rapeseed (*Brassica chinensis* L.) planting. The rhizosphere water content was maintained within 9.7–11.7%, which was more stable than that of traditional water supply and drip irrigation. As a result, microbial diversity was increased, and the dominance of *Proteobacteria*, *Acidobacteria*, etc., was eliminated from the microenvironment. Other categories of bacteria flourished, including *Actinobacteria*, *Bacteroidetes*, *Verrucomicrobia*, *Firmicutes*, *Planctomycetes*. This method provided a stable microenvironment for improving the yield and quality of rapeseed, increased the N, P, and K content in plants, and contributed to improving water use efficiency.

**TABLE 1:** Cases of water management, using CEA systems or controlled agricultural techniques to explore the impact of the microbiome on yield.

Plant	Water management method	Effects on microbial communities in rhizosphere microenvironment	Application effect	References
Tomato (*Solanum lycopersicum* L.)	Irrigation combined with aeration.	The abundance of *Acidobacteria* increased and that of *Gammaproteobacteria* decreased in response to aeration treatments; conversely, *Geobacteraceae* and *Halanaerobiaceae* were eliminated.	The ACE, Chao index, Shannon diversity index, root length, surface area, tips, and activity all increased.	Li Y. et al. (2020)
Tomato (*Solanum lycopersicum* L.)	Subsurface drip irrigation combined with concentrated organic fertilizer application.	Higher mycorrhizal colonization rates, higher abundance of microbial N-cycling genes, and lower activities of carbon-degrading enzymes were found in the rhizosphere of surface drip irrigation plants compared to full irrigation.	Tomato plants produced shorter and finer root systems with higher densities of roots around the drip line, stems and leaves increased, however, marketable tomato yield decreased by 28.3%.	Li M. et al. (2020)
Maize (*Zea mays* L.)	Well-watered irrigation and water-stressed irrigation in field blocks.	Highly significant differences (approximately 2.6–3.9% of the variation in microbial community composition) were found due to water stress. Water stress-induced belowground bacterial and archaeal microbiomes dramatically change, which were relative abundance increase of *Actinobacteria* and *Saccharibacteria* in rhizosphere, and decrease of *Chloroflexi*, *Proteobacteria*, and *Cyanobacteria*.	Water-stressed irrigation significantly reduced maize growth and productivity, among which 28% reduction was found in grain yield as compared to well-water conditions.	Wang et al. (2020)
Rapeseed (*Brassica chinensis* L.)	Supplying water based on plant consumption by using negative pressure irrigation technique.	The dominance of *Proteobacteria* and *Acidobacteria* in the rhizosphere was eliminated, and other taxa thrive, including *Actinobacteria*, *Bacteroidetes*, *Verrucomicrobia*, *Firmicutes*, *Planctomycetes*, etc.	The yield and quality of rapeseed were improved, the content of nitrogen (N), phosphorus (P), and potassium (K) of the plant was increased, and the water consumption was reduced.	Zhao et al. (2019)
Bell pepper (*Capsicum annuum* cv. Revolution)	Drip irrigation (below ground surface) subjected to well-watered and deficit irrigation levels.	Extra moisture positively induced fungi abundance through improvement in plant aboveground performance. Microbial activity at the community level decreased with water content reduction. Bacteria were more sensitive to water input changes as compared to fungi.	Higher water input contributed to the increase of pepper yield but negatively affected substrate respiration. Deficit irrigation reduced yield by 12.0% compared to the well-watered treatment, while root responses also followed a similar pattern as fruit yield.	Qin et al. (2019)
Tomato (*Solanum lycopersicum* L.)	Surface drip irrigation combined with aeration.	Aeration slightly increased mean values of the abundance of bacteria, fungi, and actinomycetes, with average increases of 4.6, 5.5, and 3.4%, respectively, and the abundance increased as irrigation amount increased.	Total root length was significantly increased by 22.2% on average under aeration, meanwhile, total root surface area and volume under the aeration was 6.6% and 6.7% higher than that of the control, respectively. Dry biomass of tomato leaf, stem, fruit, and root increased as irrigation amount increased, and the effect was significant on leaf, fruit, and root.	Chen et al. (2019a)
Maize (*Zea mays* L.)	Two levels of water stress irrigation for pot experiment.	Soil pH was lower in the rhizosphere than bulk soil but was not affected by water deficiency.	Water stress significantly decreased the rhizosphere protease activity at elongation, tasseling and grain-filling stages, and reduced the rhizosphere alkaline phosphatase activity at tasseling and grain-filling stages.	Song et al. (2012)

Indeed, the water and the air in the microenvironment are linked. To explore the impact of aeration on the microbial community, Li Y. et al. (2020) conducted an artificial aeration experiment in the soil matrix and found that an aeration treatment increased the abundance of *Acidobacteria*, reduced the abundance of *Gammaproteobacteria*, and eliminated *Geobacteraceae* and *Halanaerobiaceae*. Studies have described that *Geobacteraceae* and *Halanaerobiaceae* are closely related to *Xanthomonas*, which is an important plant pathogen (Zhang et al., 2017; Li Y. et al., 2020). The aeration improved the connection of pores, which led to a decrease in solute transport capacity and nutrient availability. It must be noted that *Acidobacteria* is an oligotrophic bacteria, which is good at reproducing with low-carbon availability (Fierer et al., 2007). The *Acidobacteria* can participate in the biogeochemical cycle, exhibit metabolic activity, and finally dominate in number under such circumstances (Lee et al., 2008). Through artificial regulation, a better rhizosphere microenvironment is created, which increases ACE, Chao index, Shannon diversity index, root length, surface area, root tip, and activity.

However, it needs to be emphasized that proper water management is more than water saving. Li M. et al. (2020) compared two different irrigation methods, namely, subsurface drip irrigation and furrow irrigation, and found that, in the rhizosphere of drip irrigation, 28.3% of the tomato (*Solanum lycopersicum* L.) yield decreased. Water limitation induced a decrease in the potential activity of carbon cycle extracellular enzymes; meanwhile, an increase in the overall abundance of microbial functional genes was involved in the N cycle process. As a result, the carbon-to-N ratio was altered in the rhizosphere microenvironment. Furthermore, water stress increased the colonization of arbuscular mycorrhizal fungi, increasing root density. Finally, the biomass of tomato plants was allocated to a non-yielding part. Therefore, although water can be used to change the interaction between plants and microbes and root morphological traits, if there are mismatches in plant demand, resource availability, and microbial carbon-to-N ratios, the goal of sustainability for CEA will not be achieved.

### Microbial Traits for Microenvironment Interaction

**FIGURE 7:**
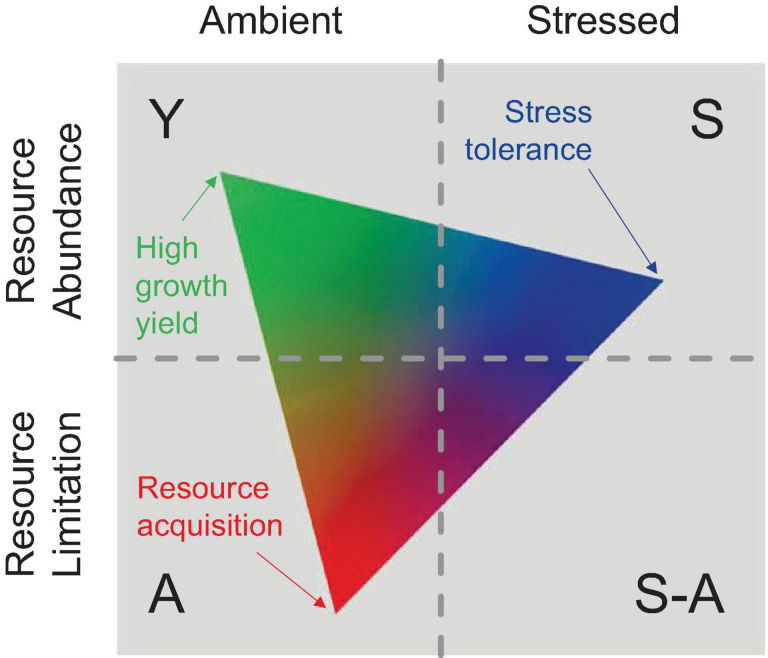
A conceptual figure of the Y-A-S framework. The Y-A-S triangle was arrayed on the combinations and represented a hypothesized particular system. The meaning of the three strategies, including the high yield (Y: maximizes growth efficiency as a result of reduced investments in stress tolerance and resource acquisition), the resource acquisition (A: preferential investment for optimized resource acquisition), and stress tolerance (S: preferential investment in stress tolerance mechanisms) (Malik et al., 2020).

Currently, there is a lack of models that characterize the microbial traits in CEA systems. The relationships between the rhizosphere competitor, stress tolerator, and ruderal can be characterized by the C-S-R framework (Grime, 1977), and the C-S-R life history triangle is a good start in advancing trait-based microbial ecology; however, it does not map well on microbes (Malik et al., 2020). Global-scale research may shed light on this issue, such as earth system models (ESMs) (Wieder et al., 2013), but they exhibited high uncertainties because they omitted key biogeochemical mechanisms (Conant et al., 2011; Schmidt et al., 2011). Microbial traits mainly correlate with resource utilization with different strategies (Hall et al., 2018; Malik et al., 2020), and thereby influence the microenvironment. The MIcrobial-MIneral Carbon Stabilization (MIMICS) model incorporated copiotrophic and oligotrophic microbial functional groups and raised hypotheses involving the roles of substrate availability, community-level enzyme induction, and microbial physiological responses (Wieder et al., 2015). Several recent efforts have applied this framework to microbial systems, particularly in the context of anthropogenic-controlled environments (Ho et al., 2013; Krause et al., 2014; Fierer, 2017; Wood et al., 2018). Malik et al. (2020) reclassified multiple factors into three main microbial strategies, which were high yield (Y), resource acquisition (A), and stress tolerance (S), and conceptualized as the Y-A-S framework (Figure 7).

Since we have reviewed much of the impact of water content variation on the all-round characteristics of CEA in this study, it is more important to conceptualize trait-based microbial strategies so that system investment could be estimated. For example, when we analyze the influence of water content variation on carbon cycling, we should consider the rhizosphere microenvironment traits as mentioned; these traits will interact with certain factors (root elongation, exudation, rhizodeposition, osmosis regulation, allelopathy, extracellular enzymes production, carbon decomposition, microbial community evolution, microbial residue chemistry, etc.) to determine long-term carbon storage in the substrate. If we evaluate this process under the Y-A-S framework (Malik et al., 2020), Y-strategists would contribute to rhizodeposition that can benefit substrate carbon accumulation. In contrast, A-strategies should contribute more to carbon decomposition through investment by enhancing microbial extracellular enzyme production (Schimel and Schaeffer, 2012; Kallenbach et al., 2016; Malik et al., 2019). On the other hand, S-strategists might depend on the type of stress compounds produced, such as osmolytes; meanwhile, it would contribute to root sheath elongation and microbial copiotrophic-oligotrophic functional alternation (Hsiao and Xu, 2000; Schimel et al., 2007; Bouskill et al., 2016b).

The same is true for other substance cycling/transformations in CEA systems, but the hindrance could be measuring and quantification (Malik et al., 2020). Current approaches have mostly focused on identifying taxonomic and functional responses to environmental changes. However, integration of these large datasets with process rate measurements remains a challenge, thereby making it difficult to link microbial composition and function with CEA systems (Krause et al., 2014; Rocca et al., 2015; Hall et al., 2018). Water variation could significantly affect rhizosphere respiration, microbial activity, and plant yield (Qin et al., 2019). Future frameworks could suggest the connection between water response and effect traits (de Vries et al., 2020).

## Conclusion and Future Perspectives

### Conclusion

In this review, we discussed the influence of water management on rhizosphere microenvironments in CEA systems, pointed out that water content variation affects the physicochemical properties of the rhizosphere substrate and changes the formation and availability of nutrients within it, thereby emphasizing the influences of macroelement transformation and consumption underwater content dynamics. Correspondently, the physiological processes in the rhizosphere are adaptively adjusted, which is achieved by the allelopathy of root exudates. In addition, from the perspective of microbiology, in rhizospheres, water content variation significantly affects microbial metabolism and proliferation, thereafter altering its nutrient type and community. The regulatory mechanism described above has important implications for CEA management. Water management can be used to seek advantages, avoid disadvantages, and establish a stable and replicable microbial microenvironment; furthermore, it could be one of the most important methodologies for benign output and sustainability for CEA systems.

### Future Perspectives

While there are promising findings that have come out from extensive research and production practices conducted to date, the CEA system is still far from large-scale utilization. Concepts of dealing with the artificial agricultural environment and its systematization require innovation (Sigrimis et al., 2001). On global food issues, we are not yet ready to deal with major environmental changes (Alvarado et al., 2020). We hypothesize there are at least two key points worth considering for the theoretical development of CEA, namely, data integration and modeling. In this study, we presented a brief overview of the two issues and made suggestions for future research and the modern development of agriculture.

#### Improvement of Universally Applicable Water Management Reference for Various CEA Systems: Data Integration

For traditional agriculture in different parts of the world, water resources management has unique regional characteristics that are calculated based on local hydrology (Hisdal and Tallaksen, 2003). Although many studies have been carried out for different crops in different regions and under different conditions, it is often difficult to replicate the same planting environment in practice (Amitrano et al., 2019). For the future systemization and globalization of the CEA, the currently published pieces of the literature showed a lack of descriptive benchmarks and norms; as a result, one management method is difficult to replicate, which hinders relevant key issues from being comparatively studied in different regions (Casaregola et al., 2016; Ladau and Eloe-Fadrosh, 2019).

Therefore, a universally applicable water management reference is crucial. At this stage, researchers are advised to focus on data acquisition for a series of key parameters, including water-related organic matter content, O_2_ concentration, respiration, substrate utilization rate, microbial composition, and activity of the rhizosphere microenvironment (Linn and Doran, 1984a; Williams, 2007; Zhou et al., 2014; Sierra et al., 2015). It is necessary to track the evolution process of the microenvironment under different water management strategies and pay attention to the environmental context and the development stage of multiple specific systems. Furthermore, it would be very beneficial to establish a database of modern CEA systems and share monitoring data and reports, which will help to accelerate the research and development of high-tech artificial agricultural ecology.

#### Quantification of Relationships Between Plant Physiology and Biochemistry Underwater Management: Modeling

To date, there is little evidence of coupling between rhizosphere microenvironment variation and microbial functional traits that affect plant physiology underwater content variation (de Vries et al., 2020). On the other hand, due to microenvironment complexity and a lack of descriptions of benchmarks and norms, it is difficult to replicate a CEA system under different planting backgrounds and promote management methods with excellent performance.

Based on massive data acquired from water management references worldwide (Sigrimis et al., 2001), we need to further offer a platform to discover the relationship between plant physiology and biochemistry by addressing ecological questions on microbial community composition and rhizosphere biogeochemical functions (Wieder et al., 2015). By evaluating the regulatory effect of water control, it is necessary to group microbial diversity into simplified functional groups and demonstrate how community differences may have a significant influence on rhizosphere substance transport and transformation. This model is expected to parameterize and accurately simulate the rhizosphere biogeochemical function of the CEA, thus showing how functional traits interact with water content gradients and follow perturbations, including wettability of substrate, enzyme activity, and microbial community (Sardans and Penuelas, 2005; Moore et al., 2010).

## Author Contributions

BT and CL: conceptualization. BT and XT: resources. BT and YL: writing – original draft preparation. BT, YL, YH, XY, and KL: writing – review and editing. BT: visualization. TL, CL, and LL: supervision. CL and LL: project administration. CL and LL: funding acquisition. All authors contributed to the article and approved the submitted version.

## Conflict of Interest

The authors declare that the research was conducted in the absence of any commercial or financial relationships that could be construed as a potential conflict of interest.

## Publisher’s Note

All claims expressed in this article are solely those of the authors and do not necessarily represent those of their affiliated organizations, or those of the publisher, the editors and the reviewers. Any product that may be evaluated in this article, or claim that may be made by its manufacturer, is not guaranteed or endorsed by the publisher.

## References

[B1] Abd El-Gayed S. S. Bashandy S. O. (2018). Effect of reduced irrigation and potassium fertilization on quantity and quality of Giza 95 cotton plants. *Egypt. J. Agron.* 40 71–84.

[B2] Abd El-Mageed T. A. El-Sherif A. M. A. Ali M. M. Abd El-Wahed M. H. (2017). Combined effect of deficit irrigation and potassium fertilizer on physiological response, plant water status and yield of soybean in calcareous soil. *Arch. Agron. Soil Sci.* 63 827–840. 10.1080/03650340.2016.1240363

[B3] Abd-Elrahman S. H. Taha N. M. (2018). Comparison between organic and mineral sources of potassium and their effects on potassium fractions in clay soil and productivity of potato plants under water stress conditions. *Egypt. J. Soil Sci.* 58 193–206. 10.1007/bf01834812

[B4] Acosta-Martinez V. Cotton J. Gardner T. Moore-Kucera J. Zak J. Wester D. (2014). Predominant bacterial and fungal assemblages in agricultural soils during a record drought/heat wave and linkages to enzyme activities of biogeochemical cycling. *Appl. Soil Ecol.* 84 69–82. 10.1016/j.apsoil.2014.06.005

[B5] Ahmadi K. Razavi B. S. Maharjan M. Kuzyakov Y. Kostka S. J. Carminati A. (2018). Effects of rhizosphere wettability on microbial biomass, enzyme activities and localization. *Rhizosphere* 7 35–42. 10.1016/j.rhisph.2018.06.010

[B6] Ahmadi K. Zarebanadkouki M. Ahmed M. A. Ferrarini A. Kuzyakov Y. Kostka S. J. (2017). Rhizosphere engineering: innovative improvement of root environment. *Rhizosphere* 3 176–184. 10.1016/j.rhisph.2017.04.015

[B7] Ahmed M. A. Kroener E. Benard P. Zarebanadkouki M. Kaestner A. Carminati A. (2016). Drying of mucilage causes water repellency in the rhizosphere of maize: measurements and modelling. *Plant Soil* 407 161–171. 10.1007/s11104-015-2749-1

[B8] Ahmed M. A. Zarebanadkouki M. Ahmadi K. Kroener E. Kostka S. Kaestner A. (2018). Engineering rhizosphere hydraulics: pathways to improve plant adaptation to drought. *Vadose Zone J.* 17 1–12.

[B9] Akter M. Deroo H. Kamal A. M. Kader M. A. Verhoeven E. Decock C. (2018). Impact of irrigation management on paddy soil N supply and depth distribution of abiotic drivers. *Agric. Ecosyst. Environ.* 261 12–24. 10.1016/j.agee.2018.03.015

[B10] Al-Kodmany K. (2018). The vertical farm: a review of developments and implications for the vertical city. *Buildings* 8 1–36.

[B11] Alster C. J. German D. P. Lu Y. Allison S. D. (2013). Microbial enzymatic responses to drought and to nitrogen addition in a Southern California grassland. *Soil Biol. Biochem.* 64 68–79. 10.1016/j.soilbio.2013.03.034

[B12] Alvarado K. A. Mill A. Pearce J. M. Vocaet A. Denkenberger D. (2020). Scaling of greenhouse crop production in low sunlight scenarios. *Sci. Total Environ.* 707:136012. 10.1016/j.scitotenv.2019.136012 31865076

[B13] Amato-Lourenco L. F. Buralli R. J. Ranieri G. R. Hearn A. H. Williams C. Mauad T. (2020). Building knowledge in urban agriculture: the challenges of local food production in Sao Paulo and Melbourne. *Environ. Dev. Sustain.* 23 2785–2796. 10.1007/s10668-020-00636-x

[B14] Amitrano C. Arena C. Rouphael Y. De Pascale S. De Micco V. (2019). Vapour pressure deficit: the hidden driver behind plant morphofunctional traits in controlled environments. *Ann. Appl. Biol.* 175 313–325. 10.1111/aab.12544

[B15] Arikan S. Pirlak L. (2016). Effects of plant growth promoting rhizobacteria (PGPR) on growth, yield and fruit quality of sour cherry (*Prunus cerasus* L.). *Erwerbs Obstbau* 58 221–226. 10.1007/s10341-016-0278-6

[B16] Armada E. Roldan A. Azcon R. (2014). Differential activity of autochthonous bacteria in controlling drought stress in native *Lavandula* and *Salvia* plants species under drought conditions in natural arid soil. *Microb. Ecol.* 67 410–420. 10.1007/s00248-013-0326-9 24337805

[B17] Armstrong A. Valverde A. Ramond J. Makhalanyane T. P. Jansson J. K. Hopkins D. W. (2016). Temporal dynamics of hot desert microbial communities reveal structural and functional responses to water input. *Sci. Rep.* 6:34434. 10.1038/srep34434 PMC5041089 27680878

[B18] Aung K. Jiang Y. He S. Y. (2018). The role of water in plant-microbe interactions. *Plant J.* 93 771–780. 29205604 10.1111/tpj.13795 PMC5849256

[B19] Bachar A. Al-Ashhab A. Soares M. I. M. Sklarz M. Y. Angel R. Ungar E. D. (2010). Soil microbial abundance and diversity along a low precipitation gradient. *Microb. Ecol.* 60 453–461. 10.1007/s00248-010-9727-1 20683588

[B20] Balafoutis A. Beck B. Fountas S. Vangeyte J. van der Wal T. Soto I. (2017). Precision agriculture technologies positively contributing to GHG emissions mitigation, farm productivity and economics. *Sustainability* 9:1339. 10.3390/su9081339

[B21] Banitalebi G. Mosaddeghi M. R. Shariatmadari H. (2019). Feasibility of agricultural residues and their biochars for plant growing media: physical and hydraulic properties. *Waste Manage.* 87 577–589. 10.1016/j.wasman.2019.02.034 31109558

[B22] Bao Q. Xiao K. Chen Z. Yao H. Zhu Y. (2014). Methane production and methanogenic archaeal communities in two types of paddy soil amended with different amounts of rice straw. *FEMS Microbiol. Ecol.* 88 372–385. 10.1111/1574-6941.12305 24579928

[B23] Barbosa G. L. Gadelha F. D. A. Kublik N. Proctor A. Reichelm L. Weissinger E. (2015). Comparison of land, water, and energy requirements of lettuce grown using hydroponic vs. conventional agricultural methods. *Int. J. Environ. Res. Public Health* 12 6879–6891. 10.3390/ijerph120606879 26086708 PMC4483736

[B24] Bateman E. J. Baggs E. M. (2005). Contributions of nitrification and denitrification to N2O emissions from soils at different water-filled pore space. *Biol. Fertil. Soils* 41 379–388. 10.1007/s00374-005-0858-3

[B25] Beckett P. H. T. Nafady M. H. M. (1967). Potassium-calcium exchange equilibria in soils – location of non-specific (Gapon) and specific exchange sites. *J. Soil Sci.* 18 263–281. 10.1111/j.1365-2389.1967.tb01505.x

[B26] Blazewicz S. J. Schwartz E. Firestone M. K. (2014). Growth and death of bacteria and fungi underlie rainfall-induced carbon dioxide pulses from seasonally dried soil. *Ecology* 95 1162–1172. 10.1890/13-1031.1 25000748

[B27] Boari A. Zuccari D. Vurro M. (2008). ‘Microbigation’: delivery of biological control agents through drip irrigation systems. *Irrig. Sci.* 26 101–107. 10.1007/s00271-007-0076-x

[B28] Bobille H. Fustec J. Robins R. J. Cukier C. Limami A. M. (2019). Effect of water availability on changes in root amino acids and associated rhizosphere on root exudation of amino acids in *Pisum sativum* L. *Phytochemistry* 161 75–85. 10.1016/j.phytochem.2019.01.015 30822623

[B29] Bonanomi G. Chirico G. B. Palladino M. Gaglione S. A. Crispo D. G. Lazzaro U. (2017). Combined application of photo-selective mulching films and beneficial microbes affects crop yield and irrigation water productivity in intensive farming systems. *Agric. Water Manage.* 184 104–113. 10.1016/j.agwat.2017.01.011

[B30] Bouskill N. J. Wood T. E. Baran R. Hao Z. Ye Z. Bowen B. P. (2016a). Belowground response to drought in a tropical forest soil. II. Change in microbial function impacts carbon composition. *Front. Microbiol.* 7:323. 10.3389/fmicb.2016.00323 27014243 PMC4791749

[B31] Bouskill N. J. Wood T. E. Baran R. Ye Z. Bowen B. P. Lim H. C. (2016b). Belowground response to drought in a tropical forest soil. I. Changes in microbial functional potential and metabolism. *Front. Microbiol.* 7:525. 10.3389/fmicb.2016.00525 27148214 PMC4837414

[B32] Bünemann E. K. (2015). Assessment of gross and net mineralization rates of soil organic phosphorus – a review. *Soil Biol. Biochem.* 89 82–98. 10.1016/j.soilbio.2015.06.026

[B33] Burchi G. Chessa S. Gambineri F. Kocian A. Massa D. Milazzo P. (2018). “Information technology controlled greenhouse: a system architecture,” in *Proceedings of the 2018 IoT Vertical and Topical Summit on Agriculture – Tuscany (IOT Tuscany)*, (Tuscany), 6.

[B34] Calvo O. C. Franzaring J. Schmid I. Mueller M. Brohon N. Fangmeier A. (2017). Atmospheric CO_2_ enrichment and drought stress modify root exudation of barley. *Global Change Biol.* 23 1292–1304. 10.1111/gcb.13503 27633609

[B35] Cameron K. C. Di H. J. Moir J. L. (2013). Nitrogen losses from the soil/plant system: a review. *Ann. Appl. Biol.* 162 145–173. 10.1111/aab.12014

[B36] Campo J. Jaramillo V. J. Maass J. M. (1998). Pulses of soil phosphorus availability in a Mexican tropical dry forest: effects of seasonality and level of wetting. *Oecologia* 115 167–172. 10.1007/s004420050504 28308448

[B37] Carminati A. Moradi A. B. Vetterlein D. Vontobel P. Lehmann E. Weller U. (2010). Dynamics of soil water content in the rhizosphere. *Plant Soil* 332 163–176.

[B38] Carminati A. Vetterlein D. Weller U. Vogel H. Oswald S. E. (2009). When roots lose contact. *Vadose Zone J.* 8 805–809. 10.2136/vzj2008.0147

[B39] Casaregola S. Vasilenko A. Romano P. Robert V. Ozerskaya S. Kopf A. (2016). An information system for European culture collections: the way forward. *SpringerPlus* 5 1–11. 10.1080/0960085x.2021.1889346 27386258 PMC4912550

[B40] Cavagnaro T. R. (2016). Soil moisture legacy effects: impacts on soil nutrients, plants and mycorrhizal responsiveness. *Soil Biol. Biochem.* 95 173–179. 10.1016/j.soilbio.2015.12.016

[B41] Chen H. Shang Z. Cai H. Zhu Y. (2019a). Irrigation combined with aeration promoted soil respiration through increasing soil microbes, enzymes, and crop growth in tomato fields. *Catalysts* 9:945. 10.3390/catal9110945

[B42] Chen H. Shang Z. Cai H. Zhu Y. (2019b). Response of soil N_2_O emissions to soil microbe and enzyme activities with aeration at two irrigation levels in greenhouse tomato (*Lycopersicon esculentum Mill.*) fields. *Atmosphere* 10:72. 10.3390/atmos10020072

[B43] Clucas B. Parker I. D. Feldpausch-Parker A. M. (2018). A systematic review of the relationship between urban agriculture and biodiversity. *Urban Ecosyst.* 21 635–643. 10.1007/s11252-018-0748-8

[B44] Colla G. Hoagland L. Ruzzi M. Cardarelli M. Bonini P. Canaguier R. (2017). Biostimulant action of protein hydrolysates: unraveling their effects on plant physiology and microbiome. *Front. Plant Sci.* 8:2202. 10.3389/fpls.2017.02202 29312427 PMC5744479

[B45] Conant R. T. Ryan M. G. Agren G. I. Birge H. E. Davidson E. A. Eliasson P. E. (2011). Temperature and soil organic matter decomposition rates - synthesis of current knowledge and a way forward. *Global Change Biol.* 17 3392–3404. 10.1111/j.1365-2486.2011.02496.x

[B46] Cournane F. C. McDowell R. W. Condron L. M. (2010). Effects of cattle treading and soil moisture on phosphorus and sediment losses in surface runoff from pasture. *N. Z. J. Agric. Res.* 53 365–376. 10.1080/00288233.2010.509903

[B47] Csonka L. N. (1989). Physiological and genetic responses of bacteria to osmotic-stress. *Microbiol. Rev.* 53 121–147. 10.1128/mr.53.1.121-147.1989 2651863 PMC372720

[B48] D’Alessio M. Durso L. M. Williams C. Olson C. A. Ray C. Paparozzi E. T. (2020). Applied injected air into subsurface drip irrigation: plant uptake of pharmaceuticals and soil microbial communities. *J. Environ. Eng.* 146:06019008. 10.1061/(asce)ee.1943-7870.0001655 29515898

[B49] de Vries F. T. Griffiths R. I. Knight C. G. Nicolitch O. Williams A. (2020). Harnessing rhizosphere microbiomes for drought-resilient crop production. *Science* 368 270–274. 10.1126/science.aaz5192 32299947

[B50] de Zeeuw H. van Veenhuizen R. Dubbeling M. (2011). The role of urban agriculture in building resilient cities in developing countries. *J. Agric. Sci.* 1491 153–163. 10.1017/s0021859610001279

[B51] Degrune F. Boeraeve F. Dufrene M. Cornelis J. Frey B. Hartmann M. (2019). The pedological context modulates the response of soil microbial communities to agroecological management. *Front. Ecol. Evol.* 7:261. 10.3389/fevo.2019.00261

[B52] DeLonge M. Vandecar K. L. D’Odorico P. Lawrence D. (2013). The impact of changing moisture conditions on short-term P availability in weathered soils. *Plant Soil* 365 201–209. 10.1007/s11104-012-1373-6

[B53] Despommier D. (2011). The vertical farm: controlled environment agriculture carried out in tall buildings would create greater food safety and security for large urban populations. *J. Verbrauch. Lebensm.* 6 233–236. 10.1007/s00003-010-0654-3

[B54] Dhaliwal S. S. Naresh R. K. Mandal A. Singh R. Dhaliwal M. K. (2019). Dynamics and transformations of micronutrients in agricultural soils as influenced by organic matter build-up: a review. *Environ. Sustain. Indic.* 1-2:100007. 10.1016/j.indic.2019.100007

[B55] Dhayalan V. Sudalaimuthu K. (2021). Plant growth promoting rhizobacteria in promoting sustainable agriculture. *Glob. J. Environ. Sci. Manage.* 7 1–18.

[B56] Drenovsky R. E. Vo D. Graham K. J. Scow K. M. (2004). Soil water content and organic carbon availability are major determinants of soil microbial community composition. *Microb. Ecol.* 48 424–430. 10.1007/s00248-003-1063-2 15692862

[B57] Eigenbrod C. Gruda N. (2015). Urban vegetable for food security in cities. A review. *Agron. Sustain. Dev.* 35 483–498. 10.1007/s13593-014-0273-y

[B58] Enebe M. C. Babalola O. O. (2018). The influence of plant growth-promoting rhizobacteria in plant tolerance to abiotic stress: a survival strategy. *Appl. Microbiol. Biol.* 102 7821–7835. 10.1007/s00253-018-9214-z 30030564 PMC6132541

[B59] Epie K. E. Maral E. (2018). Shoot and root biomass, phosphorus and nitrogen uptake of spring wheat grown in low phosphorus and moisture content conditions in a pot experiment. *J. Plant Nutr.* 41 2273–2280. 10.1080/01904167.2018.1510000

[B60] Farhangi M. H. Turvani M. E. van der Valk A. Carsjens G. J. (2020). High-tech urban agriculture in Amsterdam: an actor network analysis. *Sustainability* 12:3955. 10.3390/su12103955

[B61] Fatemeh K. Osman M. T. Raheleh A. J. Ezaz F. (2018). Opportunities and challenges in sustainability of vertical farming: a review. *J. Landsc. Ecol.* 11 35–60. 10.1515/jlecol-2017-0016

[B62] Fei Y. Huang Y. Yan C. Chao Y. He W. (2008). Influence of greenhouse cultivation on agricultural soil environment. *J. Agro Environ. Sci.* 27 243–247.

[B63] Fierer N. (2017). Embracing the unknown: disentangling the complexities of the soil microbiome. *Nat. Rev. Microbiol.* 15 579–590. 10.1038/nrmicro.2017.87 28824177

[B64] Fierer N. Bradford M. A. Jackson R. B. (2007). Toward an ecological classification of soil bacteria. *Ecology* 88 1354–1364. 10.1890/05-1839 17601128

[B65] Fischer H. Eckhardt K. Meyer A. Neumann G. Leinweber P. Fischer K. (2010). Rhizodeposition of maize: short-term carbon budget and composition. *J. Plant Nutr. Soil Sci.* 173 67–79. 10.1002/jpln.200800293

[B66] Fricano R. Davis C. (2019). How well is urban agriculture growing in the Southern United States? Trends and issues from the perspective of urban planners regulating urban agriculture. *J. Agric. Food Syst. Commun. Dev.* 9 31–53.

[B67] Frossard E. Condron L. M. Oberson A. Sinaj S. Fardeau J. C. (2000). Processes governing phosphorus availability in temperate soils. *J. Environ. Qual.* 29 15–23. 10.2134/jeq2000.00472425002900010003x

[B68] Gajc-Wolska J. Bujalski D. Chrzanowska A. (2008). Effect of a substrate on yielding and quality of greenhouse cucumber fruits. *J. Elementol.* 13 205–210.

[B69] Gao X. Zhang S. Zhao X. Long H. (2019). Stable water and fertilizer supply by negative pressure irrigation improve tomato production and soil bacterial communities. *SN Appl. Sci.* 1:718.

[B70] Ghiri M. N. Abtahi A. (2011). Potassium dynamics in calcareous vertisols of southern Iran. *Arid Land Res. Manag.* 25 257–274. 10.1080/15324982.2011.565857

[B71] Ghosh S. Watson A. Gonzalez-Navarro O. E. Ramirez-Gonzalez R. H. Yanes L. Mendoza-Suarez M. (2018). Speed breeding in growth chambers and glasshouses for crop breeding and model plant research. *Nat. Protoc.* 13 2944–2963. 10.1038/s41596-018-0072-z 30446746

[B72] Gleeson D. B. Mueller C. Banerjee S. Ma W. Siciliano S. D. Murphy D. V. (2010). Response of ammonia oxidizing archaea and bacteria to changing water filled pore space. *Soil Biol. Biochem.* 42 1888–1891. 10.1016/j.soilbio.2010.06.020

[B73] Grierson P. F. Adams M. A. (2000). Plant species affect acid phosphatase, ergosterol and microbial P in a Jarrah (*Eucalyptus marginata* Donn ex Sm.) forest in south-western Australia. *Soil Biol. Biochem.* 32 1817–1827. 10.1016/s0038-0717(00)00155-3

[B74] Grime J. P. (1977). Evidence for existence of three primary strategies in plants and its relevance to ecological and evolutionary theory. *Am. Nat.* 111 1169–1194. 10.1086/283244

[B75] Hachiya T. Sakakibara H. (2017). Interactions between nitrate and ammonium in their uptake, allocation, assimilation, and signaling in plants. *J. Exp. Bot.* 68 2501–2512. 28007951 10.1093/jxb/erw449

[B76] Halgamuge M. N. Bojovschi A. Fisher P. M. J. Le T. C. Adeloju S. Murphy S. (2021). Internet of things and autonomous control for vertical cultivation walls towards smart food growing: a review. *Urban For. Urban Gree.* 61:127094. 10.1016/j.ufug.2021.127094

[B77] Hall E. K. Bernhardt E. S. Bier R. L. Bradford M. A. Boot C. M. Cotner J. B. (2018). Understanding how microbiomes influence the systems they inhabit. *Nat. Microbiol.* 3 977–982. 10.1038/s41564-018-0201-z 30143799

[B78] Hamoud Y. A. Wang Z. Guo X. Shaghaleh H. Sheteiwy M. Chen S. (2019). Effect of irrigation regimes and soil texture on the potassium utilization efficiency of rice. *Agronomy* 9:100. 10.3390/agronomy9020100

[B79] Hansen J. C. Cade-Menun B. J. Strawn D. G. (2004). Phosphorus speciation in manure-amended alkaline soils. *J. Environ. Qual.* 33 1521–1527. 10.2134/jeq2004.1521 15254134

[B80] Hartman K. Tringe S. G. (2019). Interactions between plants and soil shaping the root microbiome under abiotic stress. *Biochem. J.* 476 2705–2724. 10.1042/bcj20180615 31654057 PMC6792034

[B81] Hartmann M. Brunner I. Hagedorn F. Bardgett R. D. Stierli B. Herzog C. (2017). A decade of irrigation transforms the soil microbiome of a semi-arid pine forest. *Mol. Ecol.* 26 1190–1206. 10.1111/mec.13995 28028891

[B82] He C. Manevski K. Andersen M. N. Hu C. Dong W. Li J. (2019). Abiotic mechanisms for biochar effects on soil N_2_O emission. *Int. Agrophys.* 33 537–546. 10.31545/intagr/111605

[B83] He M. Dijkstra F. A. (2014). Drought effect on plant nitrogen and phosphorus: a metaanalysis. *New Phytol.* 204 924–931. 10.1111/nph.12952 25130263

[B84] Hedenec P. Rui J. Lin Q. Yao M. Li J. Li H. (2018). Functional and phylogenetic response of soil prokaryotic community under an artificial moisture gradient. *Appl. Soil Ecol.* 124 372–378. 10.1016/j.apsoil.2017.12.009

[B85] Hemming S. de Zwart F. Elings A. Petropoulou A. Righini I. (2020). Cherry tomato production in intelligent greenhouses-sensors and AI for control of climate, irrigation, crop yield, and quality. *Sensors* 20 1–30. 10.3390/s20226430 PMC7698269 33187119

[B86] Herman D. J. Johnson K. K. Jaeger C. H. I. Schwartz E. Firestone M. K. (2006). Root influence on nitrogen mineralization and nitrification in *Avena barbata* rhizosphere soil. *Soil Sci. Soc. Am. J.* 70 1504–1511. 10.2136/sssaj2005.0113

[B87] Hisdal H. Tallaksen L. M. (2003). Estimation of regional meteorological and hydrological drought characteristics: a case study for Denmark. *J. Hydrol.* 281 230–247. 10.1016/s0022-1694(03)00233-6

[B88] Ho A. Kerckhof F. Luke C. Reim A. Krause S. Boon N. (2013). Conceptualizing functional traits and ecological characteristics of methane-oxidizing bacteria as life strategies. *Environ. Microbiol. Rep.* 5 335–345. 10.1111/j.1758-2229.2012.00370.x 23754714

[B89] Holland J. E. Bennett A. E. Newton A. C. White P. J. McKenzie B. M. George T. S. (2018). Liming impacts on soils, crops and biodiversity in the UK: a review. *Sci. Total Environ.* 610 316–332. 10.1016/j.scitotenv.2017.08.020 28806549

[B90] Holz M. Zarebanadkouki M. Kaestner A. Kuzyakov Y. Carminati A. (2018a). Rhizodeposition under drought is controlled by root growth rate and rhizosphere water content. *Plant Soil* 423 429–442. 10.1007/s11104-017-3522-4

[B91] Holz M. Zarebanadkouki M. Kuzyakov Y. Pausch J. Carminati A. (2018b). Root hairs increase rhizosphere extension and carbon input to soil. *Ann. Bot.* 121 61–69. 10.1093/aob/mcx127 29267846 PMC5786240

[B92] Hong E. Choi J. Nam W. Kang M. Jang J. (2014). Monitoring nutrient accumulation and leaching in plastic greenhouse cultivation. *Agric. Water Manage.* 146 11–23. 10.1016/j.agwat.2014.07.016

[B93] Hosseinzadeh S. Verheust Y. Bonarrigo G. Van Hulle S. (2017). Closed hydroponic systems: operational parameters, root exudates occurrence and related water treatment. *Rev. Environ. Sci. Biol.* 16 59–79. 10.1007/s11157-016-9418-6

[B94] Howard D. M. Howard P. J. A. (1993). Relationships between CO_2_ evolution, moisture-content and temperature for a range of soil types. *Soil Biol. Biochem.* 25 1537–1546. 10.1016/0038-0717(93)90008-y

[B95] Hsiao T. C. Xu L. K. (2000). Sensitivity of growth of roots versus leaves to water stress: biophysical analysis and relation to water transport. *J. Exp. Bot.* 51 1595–1616. 10.1093/jexbot/51.350.1595 11006310

[B96] Huang S. Sun Y. Yu X. Zhang W. (2016). Interactive effects of temperature and moisture on CO_2_ and CH_4_ production in a paddy soil under long-term different fertilization regimes. *Biol. Fertil. Soils* 52 285–294. 10.1007/s00374-015-1075-3

[B97] Hunold C. Sorunmu Y. Lindy R. Spatari S. Gurian P. L. (2016). Is urban agriculture financially sustainable? An exploratory study of small-scale market farming in Philadelphia, Pennsylvania. *J. Agric. Food Syst. Commun. Dev.* 7 51–67.

[B98] Igalavithana A. D. Lee S. S. Niazi N. K. Lee Y. Kim K. H. Park J. (2017). Assessment of soil health in urban agriculture: soil enzymes and microbial properties. *Sustainability* 9:310. 10.3390/su9020310

[B99] Jain A. Chakraborty J. Das S. (2020). Underlying mechanism of plant-microbe crosstalk in shaping microbial ecology of the rhizosphere. *Acta Physiol. Plant.* 42:8.

[B100] Jin K. Shen J. Ashton R. W. White R. P. Dodd I. C. Parry M. A. J. (2015). Wheat root growth responses to horizontal stratification of fertiliser in a water-limited environment. *Plant Soil* 386 77–88. 10.1007/s11104-014-2249-8

[B101] Jones D. L. Hodge A. Kuzyakov Y. (2004). Plant and mycorrhizal regulation of rhizodeposition. *New Phytol.* 163 459–480. 10.1111/j.1469-8137.2004.01130.x 33873745

[B102] Jones D. L. Nguyen C. Finlay R. D. (2009). Carbon flow in the rhizosphere: carbon trading at the soil-root interface. *Plant Soil* 321 5–33. 10.1007/s11104-009-9925-0

[B103] Kallenbach C. M. Frey S. D. Grandy A. S. (2016). Direct evidence for microbial-derived soil organic matter formation and its ecophysiological controls. *Nat. Commun.* 7:13630. 10.1038/ncomms13630 PMC5133697 27892466

[B104] Kim H. Sudduth K. A. Hummel J. W. (2009). Soil macronutrient sensing for precision agriculture. *J. Environ. Monit.* 11 1810–1824. 10.1039/b906634a 19809703

[B105] Knoth J. L. Kim S. Ettl G. J. Doty S. L. (2014). Biological nitrogen fixation and biomass accumulation within poplar clones as a result of inoculations with diazotrophic endophyte consortia. *New Phytol.* 201 599–609. 10.1111/nph.12536 24117518

[B106] Koch M. Naumann M. Pawelzik E. Gransee A. Thiel H. (2020). The importance of nutrient management for potato production part I: plant nutrition and yield. *Potato Res.* 63 97–119. 10.1007/s11540-019-09431-2

[B107] Kool D. M. Dolfing J. Wrage N. Van Groenigen J. W. (2011). Nitrifier denitrification as a distinct and significant source of nitrous oxide from soil. *Soil Biol. Biochem.* 43 174–178. 10.1016/j.soilbio.2010.09.030

[B108] Krause S. Le Roux X. Niklaus P. A. Van Bodegom P. M. Lennon J. T. Bertilsson S. (2014). Trait-based approaches for understanding microbial biodiversity and ecosystem functioning. *Front. Microbiol.* 5:251. 10.3389/fmicb.2014.00251 24904563 PMC4033906

[B109] Kuchenbuch R. Claassen N. Jungk A. (1986). Potassium availability in relation to soil-moisture. 2. Calculations by means of a mathematical simulation-model. *Plant Soil* 95 233–243. 10.1007/bf02375075

[B110] Kuramae E. E. Yergeau E. Wong L. C. Pijl A. S. van Veen J. A. Kowalchuk G. A. (2012). Soil characteristics more strongly influence soil bacterial communities than land-use type. *FEMS Microbiol. Ecol.* 79 12–24. 10.1111/j.1574-6941.2011.01192.x 22066695

[B111] Kuramae E. Gamper H. van Veen J. Kowalchuk G. (2011). Soil and plant factors driving the community of soil-borne microorganisms across chronosequences of secondary succession of chalk grasslands with a neutral pH. *FEMS Microbiol. Ecol.* 77 285–294. 10.1111/j.1574-6941.2011.01110.x 21488909

[B112] Kuzyakov Y. Blagodatskaya E. (2015). Microbial hotspots and hot moments in soil: concept & review. *Soil Biol. Biochem.* 83 184–199. 10.1016/j.soilbio.2015.01.025

[B113] Kuzyakov Y. Friedel J. K. Stahr K. (2000). Review of mechanisms and quantification of priming effects. *Soil Biol. Biochem.* 32 1485–1498. 10.1016/s0038-0717(00)00084-5

[B114] Kuzyakov Y. Raskatov A. Kaupenjohann M. (2003). Turnover and distribution of root exudates of *Zea mays*. *Plant Soil* 254 317–327.

[B115] Ladau J. Eloe-Fadrosh E. A. (2019). Spatial, temporal, and phylogenetic scales of microbial ecology. *Trends Microbiol.* 27 662–669. 10.1016/j.tim.2019.03.003 31000488

[B116] Lakhiar I. A. Gao J. Syed T. N. Chandio F. A. Buttar N. A. Qureshi W. A. (2018). Monitoring and control systems in agriculture using intelligent sensor techniques: a review of the aeroponic system. *J. Sensors* 2018:18.

[B117] Landi L. Valori F. Ascher J. Renella G. Falchini L. Nannipieri P. (2006). Root exudate effects on the bacterial communities, CO_2_ evolution, nitrogen transformations and ATP content of rhizosphere and bulk soils. *Soil Biol. Biochem.* 38 509–516. 10.1016/j.soilbio.2005.05.021

[B118] Lauber C. L. Hamady M. Knight R. Fierer N. (2009). Pyrosequencing-based assessment of soil pH as a predictor of soil bacterial community structure at the continental scale. *Appl. Environ. Microbiol.* 75 5111–5120. 10.1128/aem.00335-09 19502440 PMC2725504

[B119] Lazny R. Mirgos M. Przybyl J. L. Nowak J. S. Kunka M. Gajc-Wolska J. (2021). Effect of re-used lignite and mineral wool growing mats on plant growth, yield and fruit quality of cucumber and physical parameters of substrates in hydroponic cultivation. *Agronomy* 11:998. 10.3390/agronomy11050998

[B120] Lee S. Ka J. Cho J. (2008). Members of the phylum *Acidobacteria* are dominant and metabolically active in rhizosphere soil. *FEMS Microbiol. Lett.* 285 263–269. 10.1111/j.1574-6968.2008.01232.x 18557943

[B121] Li M. Schmidt J. E. LaHue D. G. Lazicki P. Kent A. Machmuller M. B. (2020). Impact of irrigation strategies on tomato root distribution and rhizosphere processes in an organic system. *Front. Plant Sci.* 11:360. 10.3389/fpls.2020.00360 32292412 PMC7118217

[B122] Li X. Rui J. Xiong J. Li J. He Z. Zhou J. (2014). Functional potential of soil microbial communities in the maize rhizosphere. *PLoS One* 9:e112609. 10.1371/journal.pone.0112609 25383887 PMC4226563

[B123] Li Y. Niu W. Zhang M. Wang J. Zhang Z. (2020). Artificial soil aeration increases soil bacterial diversity and tomato root performance under greenhouse conditions. *Land Degrad. Dev.* 31 1443–1461. 10.1002/ldr.3560

[B124] Linn D. M. Doran J. W. (1984a). Aerobic and anaerobic microbial-populations in no-till and plowed soils. *Soil Sci. Soc. Am. J.* 48 794–799. 10.2136/sssaj1984.03615995004800040019x

[B125] Linn D. M. Doran J. W. (1984b). Effect of water-filled pore-space on carbon-dioxide and nitrous-oxide production in tilled and nontilled soils. *Soil Sci. Soc. Am. J.* 48 1267–1272. 10.2136/sssaj1984.03615995004800060013x

[B126] Liu C. Rubaek G. H. Liu F. Andersen M. N. (2015). Effect of partial root zone drying and deficit irrigation on nitrogen and phosphorus uptake in potato. *Agric. Water Manage.* 159 66–76. 10.1016/j.agwat.2015.05.021

[B127] Long H. Zhang H. Yue X. Zhang R. (2018). Design and experiment of heavy liquid-type negative pressure valve used for negative pressure irrigation. *Trans. Chin. Soc. Agric. Eng.* 34 85–92.

[B128] Maestre F. T. Delgado-Baquerizo M. Jeffries T. C. Eldridge D. J. Ochoa V. Gozalo B. (2015). Increasing aridity reduces soil microbial diversity and abundance in global drylands. *Proc. Natl. Acad. Sci. U.S.A.* 112 15684–15689. 10.1073/pnas.1516684112 26647180 PMC4697385

[B129] Malik A. A. Martiny J. B. H. Brodie E. L. Martiny A. C. Treseder K. K. Allison S. D. (2020). Defining trait-based microbial strategies with consequences for soil carbon cycling under climate change. *ISME J.* 14 1–9. 10.1038/s41396-019-0510-0 31554911 PMC6908601

[B130] Malik A. A. Puissant J. Goodall T. Allison S. D. Griffiths R. I. (2019). Soil microbial communities with greater investment in resource acquisition have lower growth yield. *Soil Biol. Biochem.* 132 36–39. 10.1016/j.soilbio.2019.01.025

[B131] Manzoni S. Schimel J. P. Porporato A. (2012). Responses of soil microbial communities to water stress: results from a meta-analysis. *Ecology* 93 930–938. 10.1890/11-0026.1 22690643

[B132] Manzoni S. Trofymow J. A. Jackson R. B. Porporato A. (2010). Stoichiometric controls on carbon, nitrogen, and phosphorus dynamics in decomposing litter. *Ecol. Monogr.* 80 89–106. 10.1890/09-0179.1

[B133] Marchuk S. Marchuk A. (2018). Effect of applied potassium concentration on clay dispersion, hydraulic conductivity, pore structure and mineralogy of two contrasting Australian soils. *Soil Till. Res.* 182 35–44. 10.1016/j.still.2018.04.016

[B134] Marschner P. Grierson P. F. Rengel Z. (2005). Microbial community composition and functioning in the rhizosphere of three Banksia species in native woodland in Western Australia. *Appl. Soil Ecol.* 28 191–201. 10.1016/j.apsoil.2004.09.001

[B135] Miller W. D. Neubauer S. C. Anderson I. C. (2001). Effects of sea level induced disturbances on high salt marsh metabolism. *Estuaries* 24 357–367. 10.2307/1353238

[B136] Moore D. Kostka S. J. Boerth T. J. Franklin M. Ritsema C. J. Dekker L. W. (2010). The effect of soil surfactants on soil hydrological behavior, the plant growth environment, irrigation efficiency and water conservation. *J. Hydrol. Hydromech.* 58 142–148. 10.2478/v10098-010-0013-1

[B137] Napawan N. C. Townsend S. A. (2016). The landscape of urban agriculture in California’s capital. *Landsc. Res.* 41 780–794. 10.1080/01426397.2016.1151484

[B138] Naseem H. Ahsan M. Shahid M. A. Khan N. (2018). Exopolysaccharides producing rhizobacteria and their role in plant growth and drought tolerance. *J. Basic Microbiol.* 58 1009–1022. 10.1002/jobm.201800309 30183106

[B139] Naylor D. Coleman-Derr D. (2018). Drought stress and root-associated bacterial communities. *Front. Plant Sci.* 8:2223. 10.3389/fpls.2017.02223 29375600 PMC5767233

[B140] Niu G. Masabni J. (2018). Plant production in controlled environments. *Horticulturae* 4 1–4.

[B141] Nobel P. S. Cui M. Y. (1992). Hydraulic conductances of the soil, the root soil air gap, and the root – changes for desert succulents in drying soil. *J. Exp. Bot.* 43 319–326. 10.1093/jxb/43.3.319 12432039

[B142] Novosad N. Kay B. D. (2007). Water-filled microbially habitable pores: relation to denitrification. *Can. J. Soil Sci.* 87 269–280. 10.4141/s06-037

[B143] Ntinas G. K. Dannehl D. Schuch I. Rocksch T. Schmidt U. (2020). Sustainable greenhouse production with minimised carbon footprint by energy export. *Biosyst. Eng.* 189 164–178. 10.1016/j.biosystemseng.2019.11.012

[B144] Okegbe C. Price-Whelan A. Dietrich L. E. P. (2014). Redox-driven regulation of microbial community morphogenesis. *Curr. Opin. Microbiol.* 18 39–45. 10.1016/j.mib.2014.01.006 24607644 PMC3999281

[B145] Orsini F. Kahane R. Nono-Womdim R. Gianquinto G. (2013). Urban agriculture in the developing world: a review. *Agron. Sustain. Dev.* 33 695–720.

[B146] Orwin K. H. Bertram J. E. Clough T. J. Condron L. M. Sherlock R. R. O’Callaghan M. (2010). Impact of bovine urine deposition on soil microbial activity, biomass, and community structure. *Appl. Soil Ecol.* 44 89–100. 10.1016/j.apsoil.2009.10.004

[B147] Palta J. A. Gregory P. J. (1997). Drought affects the fluxes of carbon to roots and soil in C-13 pulse-labelled plants of wheat. *Soil Biol. Biochem.* 29 1395–1403. 10.1016/s0038-0717(97)00050-3

[B148] Parton W. J. Mosier A. R. Ojima D. S. Valentine D. W. Schimel D. S. Weier K. (1996). Generalized model for N_2_ and N_2_O production from nitrification and denitrification. *Global Biogeochem. Cycles* 10 401–412.

[B149] Pascault N. Ranjard L. Kaisermann A. Bachar D. Christen R. Terrat S. (2013). Stimulation of different functional groups of bacteria by various plant residues as a driver of soil priming effect. *Ecosystems* 16 810–822. 10.1007/s10021-013-9650-7

[B150] Pausch J. Kuzyakov Y. (2011). Photoassimilate allocation and dynamics of hotspots in roots visualized by C-14 phosphor imaging. *J. Plant Nutr. Soil Sci.* 174 12–19. 10.1002/jpln.200900271

[B151] Philippot L. Raaijmakers J. M. Lemanceau P. van der Putten W. H. (2013). Going back to the roots: the microbial ecology of the rhizosphere. *Nat. Rev. Microbiol.* 11 789–799. 10.1038/nrmicro3109 24056930

[B152] Pii Y. Mimmo T. Tomasi N. Terzano R. Cesco S. Crecchio C. (2015). Microbial interactions in the rhizosphere: beneficial influences of plant growth-promoting rhizobacteria on nutrient acquisition process. A review. *Biol. Fertil. Soils* 51 403–415. 10.1007/s00374-015-0996-1

[B153] Poncet C. Bresch C. Fatnassi H. Mailleret L. Bout A. Perez G. (2015). “Technological and ecological approaches to design and manage sustainable greenhouse production systems,” in *Proceedings of the 29^th^ International Horticultural Congress on Horticulture – Sustaining Lives, Livelihoods and Landscapes (IHC) / International Symposium on Innovation and New Technologies in Protected Cropping*, (Brisbane, QLD), 44–51.

[B154] Preece C. Penuelas J. (2016). Rhizodeposition under drought and consequences for soil communities and ecosystem resilience. *Plant Soil* 409 1–17. 10.1007/s11104-016-3090-z

[B155] Pretty J. (2018). Intensification for redesigned and sustainable agricultural systems. *Science* 362:908. 10.1126/science.aav0294 30467142

[B156] Prober S. M. Leff J. W. Bates S. T. Borer E. T. Firn J. Harpole W. S. (2015). Plant diversity predicts beta but not alpha diversity of soil microbes across grasslands worldwide. *Ecol. Lett.* 18 85–95. 10.1111/ele.12381 25430889

[B157] Qin H. Xing X. Tang Y. Zhu B. Wei X. Chen X. (2020). Soil moisture and activity of nitrite- and nitrous oxide-reducing microbes enhanced nitrous oxide emissions in fallow paddy soils. *Biol. Fertil. Soils* 56 53–67. 10.1007/s00374-019-01403-5

[B158] Qin K. Dong X. Jifon J. Leskovar D. I. (2019). Rhizosphere microbial biomass is affected by soil type, organic and water inputs in a bell pepper system. *Appl. Soil Ecol.* 138 80–87. 10.1016/j.apsoil.2019.02.024

[B159] Rajkumar M. Bruno L. B. Banu J. R. (2017). Alleviation of environmental stress in plants: the role of beneficial *Pseudomonas* spp. *Crit. Rev. Environ. Sci. Technol.* 47 372–407. 10.1080/10643389.2017.1318619

[B160] Rasool G. Guo X. Wang Z. Ali M. U. Chen S. Zhang S. (2020). Coupling fertigation and buried straw layer improves fertilizer use efficiency, fruit yield, and quality of greenhouse tomato. *Agric. Water Manage.* 239:106239. 10.1016/j.agwat.2020.106239

[B161] Read D. B. Gregory P. J. Bell A. E. (1999). Physical properties of axenic maize root mucilage. *Plant Soil* 211 87–91.

[B162] Recous S. Robin D. Darwis D. Mary B. (1995). Soil inorganic N availability: effect on maize residue decomposition. *Soil Biol. Biochem.* 27 1529–1538. 10.1016/0038-0717(95)00096-w

[B163] Rocca J. D. Hall E. K. Lennon J. T. Evans S. E. Waldrop M. P. Cotner J. B. (2015). Relationships between protein-encoding gene abundance and corresponding process are commonly assumed yet rarely observed. *ISME J.* 9 1693–1699. 10.1038/ismej.2014.252 25535936 PMC4511926

[B164] Salazar-Moreno R. Sánchez-Martínez A. C. López-Cruz I. L. (2020). Indicators for assessing water, energy and labor use performance in a low-tech greenhouse. *Rev. Chapingo Ser. Hortic.* 26 95–110. 10.5154/r.rchsh.2019.09.018

[B165] Sammauria R. Kumawat S. Kumawat P. Singh J. Jatwa T. K. (2020). Microbial inoculants: potential tool for sustainability of agricultural production systems. *Arch. Microbiol.* 202 677–693. 10.1007/s00203-019-01795-w 31897539

[B166] Sanaullah M. Chabbi A. Rumpel C. Kuzyakov Y. (2012). Carbon allocation in grassland communities under drought stress followed by C-14 pulse labeling. *Soil Biol. Biochem.* 55 132–139. 10.1016/j.soilbio.2012.06.004

[B167] Sandhya V. Ali S. Z. Grover M. Reddy G. Venkateswarlu B. (2010). Effect of plant growth promoting *Pseudomonas* spp. on compatible solutes, antioxidant status and plant growth of maize under drought stress. *Plant Growth Regul.* 62 21–30. 10.1007/s10725-010-9479-4

[B168] Sanye-Mengual E. Specht K. Krikser T. Venni C. Pennisi G. Orsini F. (2018). Social acceptance and perceived ecosystem services of urban agriculture in Southern Europe: the case of Bologna, Italy. *PLoS One* 13:e0200993. 10.1371/journal.pone.0200993 30208019 PMC6135350

[B169] Sardans J. Penuelas J. (2005). Drought decreases soil enzyme activity in a Mediterranean *Quercus ilex* L. forest. *Soil Biol. Biochem.* 37 455–461. 10.1016/j.soilbio.2004.08.004

[B170] Scavo A. Abbate C. Mauromicale G. (2019). Plant allelochemicals: agronomic, nutritional and ecological relevance in the soil system. *Plant Soil* 442 23–48. 10.1007/s11104-019-04190-y

[B171] Schimel J. P. Bennett J. (2004). Nitrogen mineralization: challenges of a changing paradigm. *Ecology* 85 591–602. 10.1890/03-8002

[B172] Schimel J. P. Schaeffer S. M. (2012). Microbial control over carbon cycling in soil. *Front. Microbiol.* 3:348. 10.3389/fmicb.2012.00348 23055998 PMC3458434

[B173] Schimel J. Balser T. C. Wallenstein M. (2007). Microbial stress-response physiology and its implications for ecosystem function. *Ecology* 88 1386–1394. 10.1890/06-0219 17601131

[B174] Schmidt M. W. I. Torn M. S. Abiven S. Dittmar T. Guggenberger G. Janssens I. A. (2011). Persistence of soil organic matter as an ecosystem property. *Nature* 478 49–56. 10.1038/nature10386 21979045

[B175] Shah A. Smith D. L. (2020). Flavonoids in agriculture: chemistry and roles in, biotic and abiotic stress responses, and microbial associations. *Agronomy* 10:1209. 10.3390/agronomy10081209

[B176] Shamshiri R. R. Kalantari F. Ting K. C. Thorp K. R. Hameed I. A. Weltzien C. (2018). Advances in greenhouse automation and controlled environment agriculture: a transition to plant factories and urban agriculture. *Int. J. Agric. Biol. Eng.* 11 1–22.

[B177] Sharp R. E. Poroyko V. Hejlek L. G. Spollen W. G. Springer G. K. Bohnert H. J. (2004). Root growth maintenance during water deficits: physiology to functional genomics. *J. Exp. Bot.* 55 2343–2351. 10.1093/jxb/erh276 15448181

[B178] Shi W. Yao J. Yan F. (2009). Vegetable cultivation under greenhouse conditions leads to rapid accumulation of nutrients, acidification and salinity of soils and groundwater contamination in South-Eastern China. *Nutr. Cycl. Agroecosyst.* 83 73–84. 10.1007/s10705-008-9201-3

[B179] Shuyu Y. Yirong D. Xiao Z. (2021). Research and application of agricultural internet of things technology in intelligent agriculture. *J. Phys.* 1769:012020. 10.1088/1742-6596/1769/1/012020

[B180] Sierra C. A. Trumbore S. E. Davidson E. A. Vicca S. Janssens I. (2015). Sensitivity of decomposition rates of soil organic matter with respect to simultaneous changes in temperature and moisture. *J. Adv. Model. Earth Syst.* 7 335–356. 10.1002/2014ms000358

[B181] Sigrimis N. Antsaklis P. Groumpos P. P. (2001). Advances in control of agriculture and the environment. *IEEE Control Syst. Mag.* 21 8–12. 10.1109/37.954516

[B182] Singh H. Poudel M. R. Dunn B. Fontanier C. Kakani G. (2020). Greenhouse carbon dioxide supplementation with irrigation and fertilization management of geranium and fountain grass. *Hortscience* 55 1772–1780. 10.21273/hortsci15327-20

[B183] Singh J. S. Pandey V. C. Singh D. P. (2011). Efficient soil microorganisms: a new dimension for sustainable agriculture and environmental development. *Agric. Ecosyst. Environ.* 140 339–353. 10.1016/j.agee.2011.01.017

[B184] Singh R. Glick B. R. Rathore D. (2020). Role of textile effluent fertilization with biosurfactant to sustain soil quality and nutrient availability. *J. Environ. Manage.* 268:110664. 10.1016/j.jenvman.2020.110664 32383645

[B185] Škarpa P. Hlušek J. (2012). Effect of years, fertilization and growing regions on the content and forms of potassium in soil. *J. Elementol.* 17 305–315.

[B186] Skopp J. Jawson M. D. Doran J. W. (1990). Steady-state aerobic microbial activity as a function of soil-water content. *Soil Sci. Soc. Am. J.* 54 1619–1625. 10.2136/sssaj1990.03615995005400060018x

[B187] Słowińska-Jurkiewicz A. Jaroszuk-Sierocińska M. (2011). “Horticulture substrates, structure and physical properties,” in *Encyclopedia of Agrophysics*, eds Gliński J. Horabik J. Lipiec J. (Dordrecht: Springer), 364–367. 10.1007/978-90-481-3585-1_67

[B188] Somaweera K. A. T. N. Suriyagoda L. D. B. Sirisena D. N. De Costa W. A. J. M. (2017). Growth, root adaptations, phosphorus and potassium nutrition of rice when grown under the co-limitations of phosphorus, potassium and moisture. *J. Plant Nutr.* 40 795–812. 10.1080/01904167.2016.1201497

[B189] Somma F. Hopmans J. W. Clausnitzer V. (1998). Transient three-dimensional modeling of soil water and solute transport with simultaneous root growth, root water and nutrient uptake. *Plant Soil* 202 281–293.

[B190] Song F. Han X. Zhu X. Herbert S. J. (2012). Response to water stress of soil enzymes and root exudates from drought and non-drought tolerant corn hybrids at different growth stages. *Can. J. Soil Sci.* 92 501–507. 10.4141/cjss2010-057

[B191] Sposito G. (2013). Green water and global food security. *Vadose Zone J.* 12 1–6. 10.2136/vzj2013.02.0041

[B192] Stark J. M. Firestone M. K. (1995). Mechanisms for soil moisture effects on activity of nitrifying bacteria. *Appl. Environ. Microbiol.* 61 218–221. 10.1128/aem.61.1.218-221.1995 16534906 PMC1388328

[B193] Styles D. Coxon C. (2006). Laboratory drying of organic-matter rich soils: phosphorus solubility effects, influence of soil characteristics, and consequences for environmental interpretation. *Geoderma* 136 120–135. 10.1016/j.geoderma.2006.03.017

[B194] Sun D. Bi Q. Li K. Dai P. Yu Y. Zhou W. (2018). Significance of temperature and water availability for soil phosphorus transformation and microbial community composition as affected by fertilizer sources. *Biol. Fertil. Soils* 54 229–241. 10.1007/s00374-017-1252-7

[B195] Sun D. Mullerova V. Ardestani M. M. Frouz J. (2019). Nitrogen fertilization and its legacy have inconsistent and often negative effect on plant growth in undeveloped post mining soils. *Soil Till. Res.* 195:104380. 10.1016/j.still.2019.104380

[B196] Suseela V. Conant R. T. Wallenstein M. D. Dukes J. S. (2012). Effects of soil moisture on the temperature sensitivity of heterotrophic respiration vary seasonally in an old-field climate change experiment. *Global Change Biol.* 18 336–348. 10.1111/j.1365-2486.2011.02516.x

[B197] Takahashi S. Ihara H. Karasawa T. (2016). Compost in pellet form and compost moisture content affect phosphorus fractions of soil and compost. *Soil Sci. Plant Nutr.* 62 399–404. 10.1080/00380768.2016.1198680

[B198] Tang S. Cheng W. Hu R. Guigue J. Kimani S. M. Tawaraya K. (2016). Simulating the effects of soil temperature and moisture in the off-rice season on rice straw decomposition and subsequent CH4 production during the growth season in a paddy soil. *Biol. Fertil. Soils* 52 739–748. 10.1007/s00374-016-1114-8

[B199] Tate R. L. (1979). Effect of flooding on microbial activities in organic soils - carbon metabolism. *Soil Sci.* 128 267–273. 10.1097/00010694-197911000-00002

[B200] Teijeiro R. G. Belimov A. A. Dodd I. C. (2020). Microbial inoculum development for ameliorating crop drought stress: a case study of *Variovorax paradoxus* 5C-2. *New Biotechnol.* 56 103–113. 10.1016/j.nbt.2019.12.006 31899322

[B201] Tocheva E. I. Ortega D. R. Jensen G. J. (2016). Sporulation, bacterial cell envelopes and the origin of life. *Nat. Rev. Microbiol.* 14 535–542. 10.1038/nrmicro.2016.85 28232669 PMC5327842

[B202] Treseder K. K. Kivlin S. N. Hawkes C. V. (2011). Evolutionary trade-offs among decomposers determine responses to nitrogen enrichment. *Ecol. Lett.* 14 933–938. 10.1111/j.1461-0248.2011.01650.x 21749597

[B203] Tuzel Y. Oztekin G. B. (2016). “Recent developments of vegetables protected cultivation in Turkey,” in *Proceedings of the 6^th^ Balkan Symposium on Vegetables and Potatoes*, (Zagreb), 435–441. 10.17660/actahortic.2016.1142.66

[B204] Ullah A. Nisar M. Ali H. Hazrat A. Hayat K. Keerio A. A. (2019). Drought tolerance improvement in plants: an endophytic bacterial approach. *Appl. Microbiol. Biol.* 103 7385–7397. 10.1007/s00253-019-10045-4 31375881

[B205] Vandecar K. L. Lawrence D. Clark D. (2011). Phosphorus sorption dynamics of anion exchange resin membranes in tropical rain forest soils. *Soil Sci. Soc. Am. J.* 75 1520–1529. 10.2136/sssaj2010.0390

[B206] Vetterlein D. Jahn R. (2004). Gradients in soil solution composition between bulk soil and rhizosphere – in situ measurement with changing soil water content. *Plant Soil* 258 307–317. 10.1023/b:plso.0000016560.84772.d1

[B207] Videgain-Marco M. Marco-Montori P. Marti-Dalmau C. Del Carmen Jaizme-Vega M. Josep Manya-Cervello J. Javier Garcia-Ramos F. (2020). Effects of biochar application in a sorghum crop under greenhouse conditions: growth parameters and physicochemical fertility. *Agronomy* 10:104. 10.3390/agronomy10010104

[B208] Vurukonda S. S. K. P. Vardharajula S. Shrivastava M. SkZ A. (2016). Enhancement of drought stress tolerance in crops by plant growth promoting rhizobacteria. *Microbiol. Res.* 184 13–24. 10.1016/j.micres.2015.12.003 26856449

[B209] Wang F. L. Huang P. M. (2001). Effects of organic matter on the rate of potassium adsorption by soils. *Can. J. Soil Sci.* 81 325–330. 10.4141/s00-069

[B210] Wang H. Boutton T. W. Xu W. Hu G. Jiang P. Bai E. (2015). Quality of fresh organic matter affects priming of soil organic matter and substrate utilization patterns of microbes. *Sci. Rep.* 5:10102. 10.1038/srep10102 PMC4426597 25960162

[B211] Wang H. Wu L. Cheng M. Fan J. Zhang F. Zou Y. (2018). Coupling effects of water and fertilizer on yield, water and fertilizer use efficiency of drip-fertigated cotton in northern Xinjiang, China. *Field Crop Res.* 219 169–179. 10.1016/j.fcr.2018.02.002

[B212] Wang P. Marsh E. L. Kruger G. Lorenz A. Schachtman D. P. (2020). Belowground microbial communities respond to water deficit and are shaped by decades of maize hybrid breeding. *Environ. Microbiol.* 22 889–904. 10.1111/1462-2920.14701 31163094

[B213] Wang Q. Xu J. Lin H. Zou P. Jiang L. (2017). Effect of rice planting on the nutrient accumulation and transfer in soils under plastic greenhouse vegetable-rice rotation system in southeast China. *J. Soil Sediment.* 17 204–209. 10.1007/s11368-016-1495-1

[B214] Welsh D. T. (2000). Ecological significance of compatible solute accumulation by micro-organisms: from single cells to global climate. *FEMS Microbiol. Rev.* 24 263–290. 10.1111/j.1574-6976.2000.tb00542.x 10841973

[B215] Wieder W. R. Bonan G. B. Allison S. D. (2013). Global soil carbon projections are improved by modelling microbial processes. *Nat. Clim. Change* 3 909–912. 10.1038/nclimate1951

[B216] Wieder W. R. Grandy A. S. Kallenbach C. M. Taylor P. G. Bonan G. B. (2015). Representing life in the earth system with soil microbial functional traits in the MIMICS model. *Geosci. Model Dev.* 8 1789–1808. 10.5194/gmd-8-1789-2015

[B217] Williams A. de Vries F. T. (2020). Plant root exudation under drought: implications for ecosystem functioning. *New Phytol.* 225 1899–1905. 10.1111/nph.16223 31571220

[B218] Williams M. A. (2007). Response of microbial communities to water stress in irrigated and drought-prone tallgrass prairie soils. *Soil Biol. Biochem.* 39 2750–2757. 10.1016/j.soilbio.2007.05.025

[B219] Wilson J. S. Baldwin D. S. Rees G. N. Wilson B. P. (2011). The effects of short-term inundation on carbon dynamics, microbial community structure and microbial activity in floodplain soil. *River Res. Appl.* 27 213–225. 10.1002/rra.1352

[B220] Witteveen C. F. B. Visser J. (1995). Polyol pools in *Aspergillus-niger*. *FEMS Microbiol. Lett.* 134 57–62. 10.1111/j.1574-6968.1995.tb07914.x 8593956

[B221] Wolf A. B. Vos M. de Boer W. Kowalchuk G. A. (2013). Impact of matric potential and pore size distribution on growth dynamics of filamentous and non-filamentous soil bacteria. *PLoS One* 8:e83661. 10.1371/journal.pone.0083661 24391805 PMC3877067

[B222] Wood J. L. Tang C. Franks A. E. (2018). Competitive traits are more important than stress-tolerance traits in a cadmium-contaminated rhizosphere: a role for trait theory in microbial ecology. *Front. Microbiol.* 9:121. 10.3389/fmicb.2018.00121 29483898 PMC5816036

[B223] Wrage N. Velthof G. L. van Beusichem M. L. Oenema O. (2001). Role of nitrifier denitrification in the production of nitrous oxide. *Soil Biol. Biochem.* 33 1723–1732. 10.1016/s0038-0717(01)00096-7

[B224] Xiao Y. Jiang S. C. Wang X. Muhammad T. Song P. Zhou B. (2020). Mitigation of biofouling in agricultural water distribution systems with nanobubbles. *Environ. Int.* 141:105787. 10.1016/j.envint.2020.105787 32402981

[B225] Xie H. Gao M. Zhang R. Xu H. Wang Y. Deng J. (2017). The subversive idea and its key technical prospect on underground ecological city and ecosystem. *Chin. J. Rock Mech. Eng.* 36 1301–1313.

[B226] Xu X. Du X. Wang F. Sha J. Chen Q. Tian G. (2020). Effects of potassium levels on plant growth, accumulation and distribution of carbon, and nitrate metabolism in apple dwarf rootstock seedlings. *Front. Plant Sci.* 11:904. 10.3389/fpls.2020.00904 32655607 PMC7325393

[B227] Yadav V. K. Raghav M. Sharma S. K. Bhagat N. (2020). Rhizobacteriome: promising candidate for conferring drought tolerance in crops. *J. Pure Appl. Microbiol.* 14 73–92. 10.22207/jpam.14.1.10

[B228] Yang J. Li Z. Liang Y. Zhang L. Li W. (2009). Effects and their mechanisms of temperature and moisture on phosphorous transformation in black soil manured with different fertilizers. *Plant Nutr. Fertil. Sci.* 15 1295–1302.

[B229] Yao Y. Yao X. An L. Bai Y. Xie D. Wu K. (2020). Rhizosphere bacterial community response to continuous cropping of Tibetan barley. *Front. Microbiol.* 11:551444. 10.3389/fmicb.2020.551444 33329420 PMC7734106

[B230] Yuan J. Zhao J. Wen T. Zhao M. Li R. Goossens P. (2018). Root exudates drive the soil-borne legacy of aboveground pathogen infection. *Microbiome* 6:156. 10.1186/s40168-018-0537-x PMC6136170 30208962

[B231] Yuste J. C. Baldocchi D. D. Gershenson A. Goldstein A. Misson L. Wong S. (2007). Microbial soil respiration and its dependency on carbon inputs, soil temperature and moisture. *Global Change Biol.* 13 2018–2035. 10.1111/j.1365-2486.2007.01415.x

[B232] Zarebanadkouki M. Carminati A. (2014). Reduced root water uptake after drying and rewetting. *J. Plant Nutr. Soil Sci.* 177 227–236. 10.1002/jpln.201300249

[B233] Zhang R. Chen L. Niu Z. Song S. Zhao Y. (2019). Water stress affects the frequency of Firmicutes, Clostridiales and Lysobacter in rhizosphere soils of greenhouse grape. *Agric. Water Manage.* 226:105776. 10.1016/j.agwat.2019.105776

[B234] Zhang X. Zhang Q. Liang B. Li J. (2017). Changes in the abundance and structure of bacterial communities in the greenhouse tomato cultivation system under long-term fertilization treatments. *Appl. Soil Ecol.* 121 82–89. 10.1016/j.apsoil.2017.08.016

[B235] Zhang Y. Hou W. Chi M. Sun Y. An J. Yu N. (2020). Simulating the effects of soil temperature and soil moisture on CO_2_ and CH_4_ emissions in rice straw-enriched paddy soil. *Catena* 194:104677. 10.1016/j.catena.2020.104677

[B236] Zhao X. Gao X. Zhang S. Long H. (2019). Improving the growth of rapeseed (*Brassica chinensis* L.) and the composition of rhizosphere bacterial communities through negative pressure irrigation. *Water Air Soil Pollut.* 230:9.

[B237] Zhou W. Hui D. Shen W. (2014). Effects of soil moisture on the temperature sensitivity of soil heterotrophic respiration: a laboratory incubation study. *PLoS One* 9:e92531. 10.1371/journal.pone.0092531 24647610 PMC3960259

[B238] Zhu X. Burger M. Doane T. A. Horwath W. R. (2013). Ammonia oxidation pathways and nitrifier denitrification are significant sources of N_2_O and NO under low oxygen availability. *Proc. Natl. Acad. Sci. U.S.A.* 110 6328–6333. 10.1073/pnas.1219993110 23576736 PMC3631630

[B239] Zulfiqar F. Shang J. Yasmeen S. Wattoo M. U. Nasrullah M. Alam Q. (2020). Urban agriculture can transform the sustainable food security for urban dwellers in Pakistan. *GeoJournal* 10.1007/s10708-020-10208-1

