# Supplementary material for: Response of Plant Rhizosphere Microenvironment to Water Management in Soil- and Substrate-Based Controlled Environment Agriculture (CEA) Systems: A Review
Source: Front Plant Sci. 2021 Aug 11;12:691651. doi: 10.3389/fpls.2021.691651 (PMC8385539; doi:10.3389/fpls.2021.691651)
Supplement: Supplementary file 1 [file Table_1.docx]

**Supplementary materials**

**Response of plant rhizosphere microenvironment to water management in** **soil- and substrate-based controlled environment agriculture (CEA) systems: a review**

Bo Tan^a^, Yihan Li^a^, Tiegang Liu^a^, Xiao Tan^a^, Yuxin He^a^, Xueji You^b,c^, Kah Hon Leong^d^, Chao Liu^a^*, Longguo Li^a^*

^a^*State Key Laboratory of Hydraulics and Mountain River Engineering,* *College of Water Resource & Hydropower, Sichuan University,* *610065, Chengdu, Sichuan, China.*

^b^*Department of Hydraulic Engineering, College of Civil Engineering, Tongji University, 1239 Siping Road, Shanghai, 200092, China.*

^c^*Department of Civil, Architectural, and Environmental Engineering, The University of Texas at Austin, 301 E. Dean Keeton St., Stop C1786, Austin, TX 78712, USA.*

^d^*Department of Environmental Engineering, Faculty of Engineering and Green Technology, Universiti Tunku Abdul Rahman, Jalan Universiti, Bandar Barat, 31900, Kampar, Perak, Malaysia.*

***Corresponding Author:**

Chao Liu (E-mail: [liuchao@scu.edu.cn](mailto:liuchao@scu.edu.cn))

Longguo Li (E-mail: [lilongguo@scu.edu.cn](mailto:lilongguo@scu.edu.cn))

**Literature Retrieval Report**

The article reviewed the interactive mechanism of water management with rhizosphere microenvironment from perspectives of physicochemical properties, physiological processes, and microbiology. In this paper, literatures presented a synthesis of relevant researches on water-root-microbes interplay, aimed to provide with detailed references to conceptualization, research, diagnosis and troubleshooting for the CEA system, and attempted to give suggestions on construction of artificial high-tech agricultural ecology. The literature information collection is of central importance. Herein, we added the detailed information on the keywords used and how they were combined for the search with query. According to the specific issues discussed in different chapters of the article, the above keywords are combined to obtain the search results. Note that some references are derived from secondary citation, which further reflect the background and basis of the research work in this article. The database: Web of Science.

For chapter 1. (Introduction) and chapter 2. (Concept and characteristics of modern CEA systems), they are introductory parts for sustainable agriculture advances, characteristics of CEA, water management and planting substrate management. Publication count vs. publication year is shown in Figure S1. The key words combination: “*controlled environment agriculture*” and “*sustainable agriculture*” or “*greenhouse cultivation*” or “urban agriculture” or “*vertical farming*” or “*sustainable greenhouse*”. When discussing specific issues, retrieval results were refined by following key words: “*ecology*”, “*irrigation* *management*”, “*growth* *promoting* *rhizobacteria*”, “*bacterial* *community*”, “*rhizosphere*”, “*rhizosphere* *microbiomes*”, “*root* *exudation*”, “*soil* *legacy*”, “*water* *treatment*”, “*water* *stress*”, “*hydroponic*”.

Figure S1 Publication count vs. publication year from 1967 to 2021 (Chapter 1 and 2). Source: Web of Science. The key words combination: “controlled environment agriculture” and “sustainable agriculture” or “greenhouse cultivation” or “urban agriculture” or “vertical farming” or “sustainable greenhouse”.

For chapter 3. (Effect of water management on availability of the CEA substrate via affecting its physiochemical properties), this part elaborated how water affects planting substrate nutrient status. Publication count vs. publication year is shown in Figure S2. The key words combination: “*controlled environment agriculture*” and “*nutrient availability*” or “*nutrient management*” or “*soil* *macronutrient*”. When discussing specific issues, retrieval results were refined by following key words: “*water* *stress*”, “*nitrogen*”, “*phosphorus*”, “*potassium*”, “*nitrification*”, “*denitrification*”, “*soil* *respiration*”, “*oxygen* *availability*”, “*microbial* *communities*”.

Figure S2 Publication count vs. publication year from 1967 to 2021 (Chapter 3). The topic key words for this chapter were combined by: “controlled environment agriculture” and “nutrient availability” or “nutrient management” or “soil macronutrient”. Retrieval results were refined by following key words: “water stress”, “nitrogen”, “phosphorus”, “potassium”, “nitrification”, “denitrification”, “soil respiration”, “oxygen availability”, “microbial communities”. Source: Web of Science.

For chapter 4. (Effect of water management on root physiological processes in CEA), this part summarized and analyzed how water affects root growth and exudation, rhizosphere allelopathy and rhizosphere physiological adaptation. Publication count vs. publication year is shown in Figure S3. The key words combination: “*controlled environment agriculture*” and “*water transport*” or “*water* *availability*” or “*soil* *water* *content*”. When discussing specific issues, retrieval results were refined by following key words: “*water* *repellency*”, “*plant* *adaptation*”, “*microbial* *communities*”, “*drought* *effect*”, “*root* *physiology*”.

Figure S3 Publication count vs. publication year from 1967 to 2021 (Chapter 4). The topic key words for this chapter were combined by: “controlled environment agriculture” and “water transport” or “water availability” or “soil water content”. Retrieval results were refined by following key words: “water repellency”, “plant adaptation”, “microbial communities”, “drought effect”, “root physiology”. Source: Web of Science.

For the chapter 5. (Effect of water management on rhizosphere microbiology in CEA), this part addressed the microbial physiology, microbial community, and microbial traits for microenvironment interaction. Publication count vs. publication year is shown in Figure S4. The key words combination: “*controlled environment agriculture*” and “*microbial* *physiology*” or “*microbial* *community*” or “*microbial* *diversity*” or “*microbial* *activities*”. When discussing specific issues, retrieval results were refined by following key words: “*microbial* *stress-response*”, “*residue* *decomposition*”, “*plant growth promoting rhizobacteria*”, “*microbial functional traits*”, “*substrate* *utilization*”, “*mineralization*”, “*soil enzymes*”, “*plant physiology*”

Figure S4 Publication count vs. publication year from 1967 to 2021 (Chapter 5). The topic key words for this chapter were combined by: “controlled environment agriculture” and “microbial physiology” or “microbial community” or “microbial diversity” or “microbial activities”. Retrieval results were refined by following key words: “microbial stress-response”, “residue decomposition”, “plant growth promoting rhizobacteria”, “microbial functional traits”, “substrate utilization”, “mineralization”, “soil enzymes”, “plant physiology”. Source: Web of Science.
